# Literature Review of Studies Using the National Database of the Health Insurance Claims of Japan (NDB): Limitations and Strategies in Using the NDB for Research

**DOI:** 10.31662/jmaj.2023-0078

**Published:** 2023-12-27

**Authors:** Maiko Suto, Arisa Iba, Takehiro Sugiyama, Tomoko Kodama, Misa Takegami, Reina Taguchi, Mariko Niino, Ryuji Koizumi, Kimikazu Kashiwagi, Kenjiro Imai, Noriko Ihana-Sugiyama, Yuichi Ichinose, Kenji Takehara, Hiroyasu Iso

**Affiliations:** 1 Department of Health Policy, National Center for Child Health and Development, Tokyo, Japan; 2 Institute for Global Health Policy Research, Bureau of International Health Cooperation, National Center for Global Health and Medicine, Tokyo, Japan; 3 Department of Health Services Research, Institute of Medicine, University of Tsukuba, Ibaraki, Japan; 4 Diabetes and Metabolism Information Center, Research Institute, National Center for Global Health and Medicine, Tokyo, Japan; 5 Department of Public Health Policy, National Institute of Public Health, Saitama, Japan; 6 Department of Preventive Medicine and Epidemiology, National Cerebral and Cardiovascular Center, Osaka, Japan; 7 Department of Public Health and Health Policy, School of Medicine, The University of Tokyo, Tokyo, Japan; 8 Institute for Health Economics and Policy, Tokyo, Japan; 9 Division of Health Services Research, Institute for Cancer Control, National Cancer Center, Tokyo, Japan; 10 AMR Clinical Reference Center, Disease Control and Prevention Center, National Center for Global Health and Medicine, Tokyo, Japan; 11 National College of Nursing, Tokyo, Japan

**Keywords:** NDB, health insurance claims, healthcare policy, Japan, review

## Abstract

The use of the National Database of Health Insurance Claims and Specific Health Checkups of Japan (NDB) for research has increased over time. Researchers need to understand the characteristics of the data to generate quality-assured evidence from the NDB. In this review, we mapped and characterized the limitations and related strategies using the NDB for research based on the descriptions of published NDB studies. To find studies that used Japanese healthcare claims data, we searched MEDLINE, EMBASE, and Ichushi-Web up to June 2023. Additionally, we hand-searched the NDB data publication list from the Ministry of Health, Labour and Welfare (2017-2023). We abstracted data based on the NDB data type, research themes, age of the study sample or population, targeted disease, and the limitations and strategies in the NDB studies. Ultimately, 267 studies were included. Overall, the most common research theme was describing and estimating the prescriptions and treatment patterns (125 studies, 46.8%). There was a variation in the frequency of themes according to the type of NDB data. We identified the following categories of limitations: (1) lack of information on confounders/covariates, outcomes, and other clinical content, (2) limitations regarding patients not included in the NDB, (3) misclassification of data, (4) lack of unique identifiers and register of beneficiaries, and (5) others. Although the included studies noted several limitations of using the NDB for research, they also provided some strategies to address them. Organizing the limitations of NDB in research and the related strategies across research fields can help support high-quality NDB studies.

## 1. Background

Administrative healthcare databases have been used in many countries for epidemiological and health services research ^(1), (2), (3), (4), (5)^. In Japan, the National Database of Health Insurance Claims and Specific Health Checkups of Japan (NDB) is a large claims database. Japan has a universal health insurance system, and the NDB includes over 98% of all claims, making it a highly comprehensive database for medical diagnoses and practices in Japan ^(6)^. The NDB data contains information such as the patient’s sex and age, the location and number of beds in medical institutions, and the type of injury or disease, medical treatment, drug administration, and prescriptions received by the insured.

In 2011, the Ministry of Health, Labour and Welfare (MHLW) began providing NDB data for research purposes. To ensure patient confidentiality and anonymity, researchers are provided with the minimum amount of NDB data necessary for their research. Depending on the research purpose, several data types are provided, which include the NDB General Data (“special extraction” data and data used via the NDB Onsite Research Center), Sampling Data, Accumulated Data, and Open Data. Researchers can apply to the MHLW to use the NDB General Data and will be provided with all the data deemed necessary for their study. For exploratory studies, researchers can follow a more simplified application process to receive and acquire the Sampling Data, which is extracted from the claims information of a single month. Furthermore, the MHLW provides Accumulated Data, which does not contain any personally identifiable information ^(6)^. Lastly, without requiring an application, the NDB Open Data are published as basic and versatile tabulation tables on the MHLW website ^(7)^.

Since its creation, there has been an increase in the use of the NDB for research purposes. However, using NDB for research has several challenges. Researchers must understand the limitations of using the NDB for research as well as the characteristics of the available data to generate quality-assured evidence. In a previous review of studies that used the NDB, the authors reported that some of the typically encountered limitations in the included studies were the lack of important information (e.g., disease severity, socioeconomic status, and family history), absence of validation studies, and difficulty in drawing causal inferences from retrospective data ^(8)^. Okumura et al. ^(9)^ introduced the pitfalls of research using health insurance claims, including uncertainties in admission, discharge dates, injury and drug information, and the problem of patients becoming untraceable due to insurance withdrawals, along with solutions based on their experiences. There are other comprehensive reviews on NDB studies ^(10), (11)^ but they do not address the limitations of using the NDB for research; therefore, the findings on the limitations in research findings from studies based on NDB data are not systematically organized. In this review, we comprehensively mapped the limitations, strategies, and characteristics of studies that used NDB based on the descriptions in the published studies to promote future NDB research that provides high-quality evidence.

## 2. Methods

### 2.1. Search and selection of sources of evidence

To identify published NDB studies, we searched MEDLINE, EMBASE, and Ichushi-Web for records up to June 2023. The search strategies for each source were developed by two experienced information specialists (complete electronic search strategies are presented in Supplementary File 1). Additionally, we hand-searched the NDB data publication list reported by the MHLW (2017-2023).

We formulated search strategies to identify studies that used health insurance claims data (not limited to the NDB). First, we extracted studies using health insurance claims data and then selected those that used NDB data (the study selection flowchart is shown in Supplementary File 2). Reviewers working in pairs independently assessed the titles and abstracts retrieved from electronic searches using Rayyan software. Each full-text screening was conducted by a reviewer who extracted data from the included studies using the data-extraction form developed for this review through a discussion. Subsequently, a reviewer confirmed the inclusion or exclusion of studies and data-extraction results throughout the study to ensure that the categories were consistent. The excluded studies in the full-text screening stage with the reasons for their exclusion are listed in Supplementary File 3.

### 2.2. Eligibility criteria

We included studies that utilized the following NDB datasets: (1) General Data (“special extraction” data and data used via the NDB Onsite Research Center), (2) Sampling Data, (3) Accumulated Data, and (4) Open Data. Studies that only used health checkup data or were not original articles (such as conference abstracts, reviews, commentaries, letters, or study notes) were excluded. The characteristics of each NDB dataset are described below.

#### 2.2.1. General data

NDB General Data are referred to as “special extraction” data. To use the data, researchers must submit their study protocols and obtain approval from the MHLW advisory committee. Upon approval, researchers are provided with the extracted data deemed necessary for their studies. The data contains anonymized identifiers and can be linked to the claims data for the same patients, enabling longitudinal analysis. Researchers can also access all the data stored in the NDB for the last 10 years via the NDB Onsite Research Center ^(12)^.

#### 2.2.2. Sampling data

Sampling Data includes randomly selected claims data covering 1% of all outpatients and 10% of all inpatients after excluding high-cost claims since 2011 in the months of January, April, July, and October. In this dataset, codes that occur <0.1% of the time (<0.01% for medical practices) are anonymized ^(13)^. Unlike General Data, these data can be obtained through a simplified review process, allowing researchers to conduct exploratory studies more easily.

#### 2.2.3. Accumulated data

Accumulated Data are provided by the MHLW as aggregated data that do not include personally identifiable information. Accumulated Data are created using no more than three axes: e.g., sex, age group, and prefecture ^(14)^.

#### 2.2.4. Open data

The Open Data is shown in a tabulation table published on the MHLW website. The first set of NDB Open Data includes the FY2014 medical service data and specific health checkups for FY2013. The seventh set of NDB Open Data, including the FY2020 medical service data and FY2019 specific health checkups, was published in December 2022. It is available on the MHLW website, and applications are not necessary. The first set of NDB Open Data includes (1) medical treatments, (2) dental disease, (3) results of checkups, and (4) drug data. The type of data provided has gradually expanded, and the seventh set of Open Data also includes (5) dental treatments, (6) prescriptions, (7) special treatment materials, and (8) questionnaires from checkups ^(7)^. The tabulation table provides statistics by prefecture, sex, and age group. The range and granularity of information have changed gradually; for example, in the seventh set of NDB Open Data, for the aggregations of target medical treatments and dental treatments, statistics were also provided for the secondary medical area and the month of medical treatment ^(7)^.

### 2.3. Data items and synthesis of results

We abstracted data based on the type of NDB dataset used (General, Sampling, Accumulated, or Open Data), research theme, age of the study sample or population, and targeted disease. For targeted diseases and research themes, we selected up to two categories per study (studies targeting more than two diseases were classified as “others”). The characteristics of the included studies are provided in Supplementary File 4.

For the limitations of using the NDB for research (such as lack of important information and absence of validation studies), categories were created through a discussion regarding previous studies ^(8), (9)^, and related information was extracted from descriptions in the “Methods” and “Discussion” sections of the included studies. Strategies for addressing each limitation were also extracted from the descriptions in those sections.

**Figure 1.**
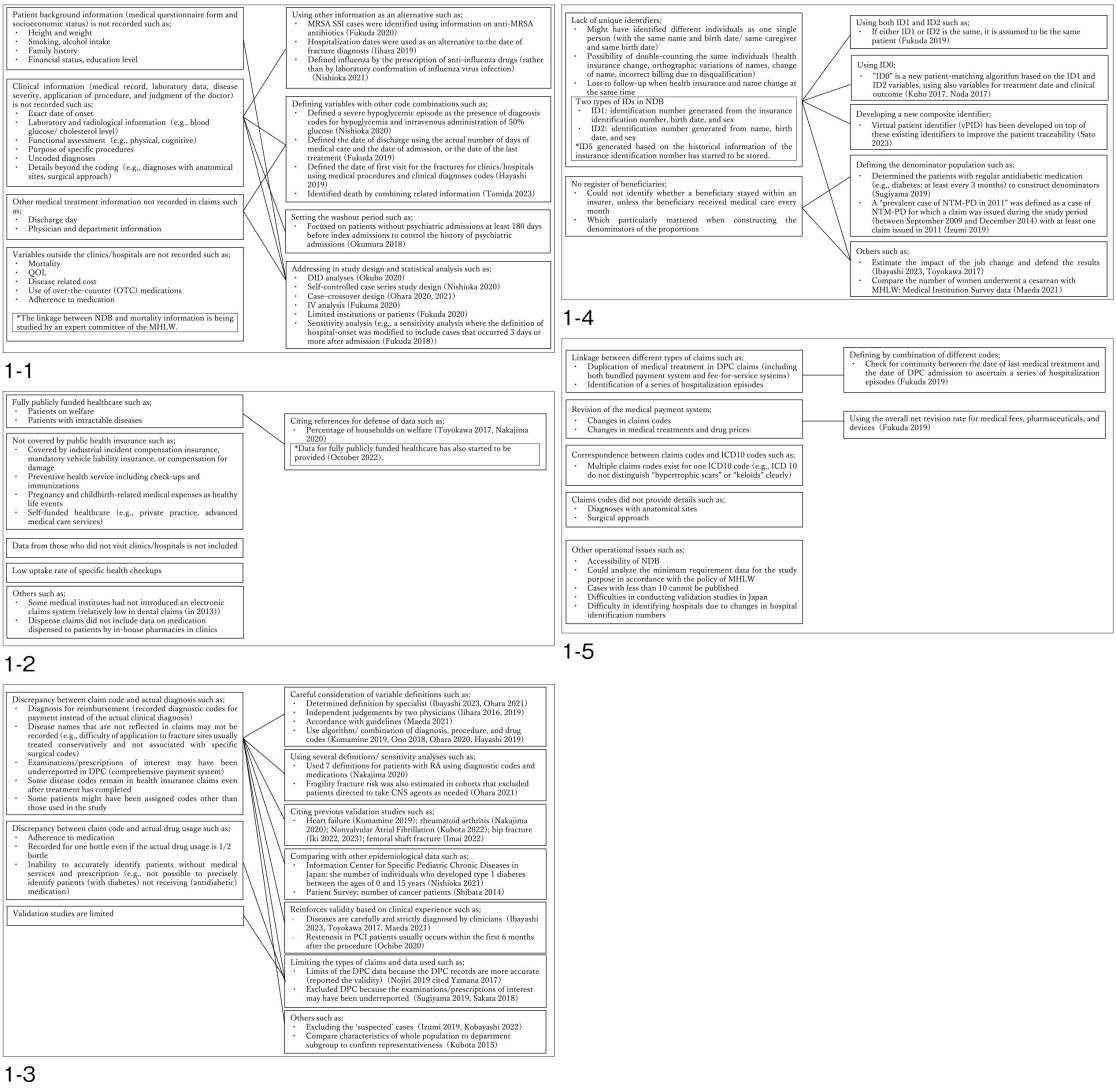
Limitations and strategies for NDB studies 1-1: Lack of information on confounders/covariates, outcomes, and other clinical content 1-2: Limitations regarding patients not included in the NDB 1-3: Misclassification of data 1-4: Lack of unique identifiers and register of beneficiaries 1-5: Other limitations *The box on the left shows the limitations, and the box on the right shows the strategies.

We summarized the study characteristics (research theme, age of the study sample, and targeted disease) based on the NDB data type (Table 1). Moreover, we categorized each limitation as well as mapped paired limitations and strategies in the NDB studies (Figure 1). Limitations specific to each data type other than General Data were narratively described in the text. We conducted this review following the PRISMA-ScR reporting guidelines (Supplementary File 5) ^(15)^.

**Table 1.** Characteristics of the Included Studies According to Data Type.

	Number of studies	All		General data		Sampling data		Accumulated data		Open data	
	Total	267	%	151	%	27	%	15	%	74	%
Theme	Medical treatment status	125	46.8	51	33.8	20	74.1	7	46.7	47	63.5
	Clinical epidemiology, course of diseases	67	25.1	40	26.5	8	29.6	8	53.3	11	14.9
	Socioeconomic comparison (e.g., prefecture)	46	17.2	12	7.9	1	3.7	3	20.0	30	40.5
	Intervention effect	45	16.9	42	27.8	1	3.7	0	0.0	2	2.7
	Research methodology	26	9.7	15	9.9	1	3.7	6	40.0	4	5.4
	Health policy evaluation and utilization	26	9.7	14	9.3	2	7.4	0	0.0	10	13.5
	Health economics	19	7.1	13	8.6	0	0.0	0	0.0	6	8.1
	Quality of care	16	6.0	8	5.3	7	25.9	0	0.0	1	1.4
	Patient health service utilization	13	4.9	10	6.6	0	0.0	0	0.0	3	4.1
	Others	10	3.7	6	4.0	3	11.1	0	0.0	1	1.4
	Prediction model	5	1.9	1	0.7	0	0.0	1	6.7	3	4.1
Age	Children	17	6.4	12	7.9	3	11.1	0	0.0	2	2.7
	Older persons	21	7.9	17	11.3	2	7.4	1	6.7	1	1.4
	Adults	66	24.7	44	29.1	5	18.5	2	13.3	15	20.3
	No age limit/Others	163	61.0	78	51.7	17	63.0	12	80.0	56	75.7
Disease	1) Certain infectious and parasitic diseases	30	11.2	18	11.9	8	29.6	2	13.3	2	2.7
	2) Neoplasms	14	5.2	5	3.3	5	18.5	1	6.7	3	4.1
	3) Diseases of the blood and blood-forming organs and certain disorders involving the immune mechanism	0	0.0	0	0.0	0	0.0	0	0.0	0	0.0
	4) Endocrine, nutritional, and metabolic diseases	25	9.4	20	13.2	1	3.7	0	0.0	4	5.4
	5) Mental, Behavioral, and Neurodevelopmental disorders	23	8.6	13	8.6	4	14.8	1	6.7	5	6.8
	6) Diseases of the nervous system	18	6.7	11	7.3	1	3.7	0	0.0	6	8.1
	7) Diseases of the eye and adnexa	5	1.9	3	2.0	1	3.7	0	0.0	1	1.4
	8) Diseases of the ear and mastoid process	0	0.0	0	0.0	0	0.0	0	0.0	0	0.0
	9) Diseases of the circulatory system	38	14.2	27	17.9	5	18.5	0	0.0	6	8.1
	10) Diseases of the respiratory system	12	4.5	7	4.6	3	11.1	2	13.3	0	0.0
	11) Diseases of the digestive system	21	7.9	11	7.3	0	0.0	0	0.0	10	13.5
	12) Diseases of the skin and subcutaneous tissue	4	1.5	3	2.0	0	0.0	0	0.0	1	1.4
	13) Diseases of the musculoskeletal system and connective tissue	30	11.2	21	13.9	0	0.0	1	6.7	8	10.8
	14) Diseases of the genitourinary system	9	3.4	4	2.6	2	7.4	0	0.0	3	4.1
	15) Pregnancy, childbirth, and the puerperium	5	1.9	3	2.0	1	3.7	1	6.7	0	0.0
	16) Certain conditions originating in the perinatal period	1	0.4	0	0.0	0	0.0	0	0.0	1	1.4
	17) Congenital malformations, deformations, and chromosomal abnormalities	4	1.5	3	2.0	0	0.0	0	0.0	1	1.4
	18) Injury, poisoning, and certain other consequences of external causes	29	10.9	20	13.2	2	7.4	2	13.3	5	6.8
	19) Others (multidisease, not focused on specific diseases)	53	19.9	21	13.9	3	11.1	6	40.0	23	31.1
^*^Theme and disease: up to two categories are selected										

## 3. Results

### 3.1. Characteristics of included studies

We identified 267 studies through electronic searches and a hand search of the NDB’s publication list. Among the 267 articles included in the review, 151 used General Data (some studies used the NDB Onsite Research Center ^(16), (17), (18)^), 27 used Sampling Data, 15 used Accumulated Data, and 74 used Open Data. Table 1 shows the characteristics of the included studies by NDB data type.

Overall, the most common research theme, regardless of NDB data type, was describing and estimating prescriptions and treatment patterns (125 studies: 46.8%), followed by clinical epidemiology and the course of specific diseases, such as the number of patients, incidence, risk factors, and related factors (67 studies: 25.1%). By data type, a relatively large number of studies examined intervention effects, including the effectiveness and adverse effects of treatments and drugs using General Data (42 out of 151 General Data studies: 27.8%). For Sampling Data, many studies were conducted to determine medical treatment status (20 out of 27 Sampling Data studies: 74.1%). Regarding Accumulated Data, several studies focused on research methodology, comparing the NDB with other data sources, including estimates of drug use or patients (6 out of 15 Accumulated Data studies: 40.0%). For Open Data, many studies focused on socioeconomic comparisons, including sex, age, and regional differences in disease and treatment patterns (30 out of 74 Open Data studies: 40.5%).

Regarding targeted diseases, most studies focused on diseases of the circulatory system (38 studies: 14.2%); infectious diseases (30 studies: 11.2%); diseases of the musculoskeletal system and connective tissue (30 studies: 11.2%); injury, poisoning, and other consequences from external causes (29 studies: 10.9%, 20 studies on fractures); endocrine, nutritional, and metabolic diseases (25 studies: 9.4%, 21 studies on diabetes); psychiatric diseases (23 studies: 8.6%), and diseases of the digestive system (21 studies: 7.9%, 14 studies on dental diseases). By age group, respiratory diseases and influenza were more prevalent among pediatric populations, while fractures were more prevalent among older adults (Table 1, Supplementary File 4).

### 3.2. Limitations and strategies in the NDB studies

We extracted information about the limitations and strategies of using the NDB for research from the descriptions in the “Methods” and “Discussion” sections of each included study. The following categories of limitations were identified: (1) lack of information on confounders/covariates, outcomes, and other clinical content; (2) limitations regarding patients not included in the NDB; (3) misclassification of data; (4) lack of unique identifiers and registers of beneficiaries; and (5) others. The limitations and their strategies are mapped in Figure 1. In addition, the limitations specific to each type of data other than General Data are narratively described.

#### 3.2.1. Issue 1: Lack of information on confounders/covariates, outcomes, and other clinical content

The first limitation is the need for more important information for research related to confounders/covariates, outcomes, and other clinical content. Patients’ background information, such as their answers in medical questionnaires and their socioeconomic status, as well as their clinical information, such as medical records, laboratory data, disease severity, application of procedures, and judgment of the doctor, are not recorded in the NDB. In addition, it is impossible to determine variables outside the hospital or clinic, such as mortality, quality of life, and medication adherence.

To overcome these limitations, the following strategies were reported: (1) using other information as an alternative, such as defining influenza based on the prescription of anti-influenza drugs rather than by laboratory confirmation of an infection with the influenza virus ^(19)^; (2) defining variables based on other code combinations, such as defining a severe hypoglycemic episode as the presence of a diagnosis code for hypoglycemia and an intravenous administration of 50% glucose ^(20)^; (3) setting a washout period, such as excluding patients who were admitted to any psychiatric unit within 180 days before the index admission to control for a history of psychiatric admissions ^(21)^; and (4) addressing the study design and statistical analysis, such as difference-in-difference analyses ^(22)^, self-controlled case-series study design ^(23)^, case-crossover design ^(24), (25)^, and instrumental variable analysis ^(26)^.

#### 3.2.2. Issue 2: Limitations regarding patients not included in the NDB

The NDB does not include claims data from specific patient populations, including those with fully publicly funded healthcare* (e.g., patients with intractable diseases and patients on welfare) (*data for fully publicly funded healthcare were not provided until 2022) ^(27)^ and those not covered by the public health insurance (e.g., covered by industrial incident compensation insurance, mandatory vehicle liability insurance, or compensation for damage within preventive health services including checkups and immunizations, pregnancy and childbirth-related medical expenses, as well as self-funded healthcare). In addition, the NDB does not include data from patients who did not visit clinics or hospitals. Further, the rate of specific health checkups has been low ^(28)^. Some medical institutes have not introduced an electronic claims system (relatively low for dental claims compared other medical claims in 2013 ^(29)^; the incomplete coverage of dental claims is due to the incomplete adoption of an electronic claims system, which decreased until 2015 and then stabilized ^(6)^), and their data have not been included in the NDB.

Few strategies are available to address these limitations. Some studies have cited reference data, such as the percentage of households on welfare, to show how small the impact was on their outcomes ^(30), (31)^.

#### 3.2.3. Issue 3: Misclassification of data

NDB data for injury or disease, treatment, drug administration, and prescriptions are based on claims codes. Linking NDB data to other databases is not permitted, making it difficult to conduct validation studies. Discrepancies can occur between claim codes and actual diagnoses, such as recording diagnostic codes for payment instead of the actual clinical diagnoses, not recording disease names and examinations or prescriptions that are not reflected in the claims, and disease codes remaining in the health insurance claims even after treatment has been completed. Additionally, there were discrepancies between claim codes and actual drug usage, making it difficult to accurately ascertain the amount of drugs used ^(32)^.

The limitations were overcome by the following strategies: (1) careful consideration of variable definitions (e.g., definitions determined by specialists ^(25), (33)^, independent judgements ^(34), (35)^, reference to guidelines ^(36)^, and using an algorithm for defining the diagnosis, treatments, procedures, and drug codes ^(24), (37), (38), (39)^); (2) using several definitions and conducting sensitivity analyses ^(25), (31)^; (3) citing previous validation studies ^(31),(37), (40), (41), (42), (43)^; (4) making comparisons with other epidemiological data ^(19), (44)^; (5) reinforcing validity based on clinical experience (e.g., diseases are carefully and strictly diagnosed by clinicians ^(30), (33), (45)^); (6) limiting the types of claims and data used (e.g., limiting the Diagnosis Procedure Combination (DPC) data because the DPC records are more accurate ^(46), (47)^, or excluding DPC because the examinations or prescriptions of interest may have been underreported in the comprehensive payment system ^(48), (49)^); and (7) other strategies, such as excluding “suspected” cases ^(50), (51)^.

#### 3.2.4. Issue 4: Lack of unique identifiers and register of beneficiaries

The NDB does not have unique identifiers for individuals, but instead uses two types of patient identifiers made from the available claims information: ID1 is an identification number generated from the patient’s insurance identification number, birthdate, and sex, and ID2 is an identification number generated from the patient’s name, birthdate, and sex. Neither is unique, and they can change with life events; therefore, different patients could be identified as the same individual, or one patient could be identified as different individuals ^(52)^. In addition, follow-up can become difficult if a patient’s health insurance and name change simultaneously.

Furthermore, another limitation is the lack of a register of insured patients. Identifying whether beneficiaries remain with the insurer can be difficult if they do not receive medical care every month. This is particularly important when constructing denominators of proportions ^(48)^.

A strategy for overcoming these limitations is to combine ID1 and ID2 in an attempt to reduce misclassification. For example, if one of the IDs is the same, it is assumed to be the same patient ^(32)^. Several studies used “ID0,” which is a new patient-matching algorithm based on the ID1 and ID2 variables, as well as using variables for treatment date and clinical outcome ^(53), (54)^. A new composite identifier, “virtual patient identifier,” has also been developed to improve patient traceability ^(55)^. To overcome the lack of a register of beneficiaries, one study identified patients with regular antidiabetic medication (e.g., diabetes: at least every three months) to construct a denominator population ^(48)^. Some studies cited reference data to estimate the impact of a change in employment on patient ID changes ^(30), (33)^ or compare the size of the targeted population with that of government statistics ^(45)^.

#### 3.2.5. Issue 5: Others

The following limitations of the NDB were also noted: (1) issues related to the linkage between different types of claims, including identification of a series of hospitalization episodes; (2) issues related to changes in the medical payment system; (3) issues related to the correspondence between claim codes and ICD10 codes; (4) issues related to claim codes not providing certain details (e.g., diagnoses with anatomical sites and surgical approach); and (5) other operational issues, such as NDB accessibility and difficulties in conducting validation studies. To address these limitations, a method for identifying a series of hospitalization episodes by checking the continuity between the last medical treatment date and the DPC admission date, as well as a method to use the overall net revision rate, were reported ^(32), (56)^.

#### 3.2.6. Limitations specific to each data type other than general data

For Sampling Data, the specific limitations were as follows: (1) data for a single month (e.g., October) cannot account for seasonal effects and monthly trends, may miss patients who did not visit a healthcare facility in that month ^(57), (58)^, and cannot confirm the outcomes of treatment lasting longer than one month ^(59)^; (2) as a patient ID is only provided in the outpatient data, inpatient and outpatient claims for the same patient cannot be linked ^(60), (61)^ (several studies linked outpatient and prescription claims); (3) high-cost claims and rare codes are not included to prevent the identification of patients receiving rare treatments (e.g., one study reported that 27%-70% of patients who received chemotherapy drugs were anonymized) ^(61), (62)^.

For Accumulated Data, studies were conducted at the ecological level. Future studies may need to be conducted at the individual level to verify associations or causal relationships ^(63), (64)^. As it could not create complex variable definitions, detailed information, such as patients’ underlying diseases, cannot be ascertained ^(65)^.

Limitations specific to Open Data were identified as follows: (1) data regarding the top 100 drugs in each therapeutic category are disclosed, whereas the data for some rarely used drugs are not included to ensure the confidentiality and anonymity of the NDB data ^(66), (67)^; (2) the available information in the NDB Open Data is based on “prescription volume,” without information on the number of patients ^(68), (69)^ (e.g., estimating the number of patients by assuming dosage per patient ^(66)^); (3) analysis across different medical facilities is not possible (e.g., identifying patients who receive a total of seven medications or more from multiple facilities) ^(70)^; (4) issues can arise due to ecological studies (e.g., prefecture-wide data does not always apply to individual hospitals and patients) ^(71), (72), (73)^; and (5) prefecture-level data might show different characteristics from smaller regions ^(74), (75)^. The types of information provided in the Open Data have gradually expanded, and the available data items and levels of detail vary depending on the year in which the data were published.

## 4. Discussion

Many studies have used the NDB to describe and estimate disease prevalence and incidence, treatment and prescription patterns, and regional differences, as well as to establish associations or causal relationships, including risk factors, treatment effects, and adverse events. Moreover, several studies on common diseases, such as infectious diseases, influenza, pneumonia, diabetes, psychiatric diseases, cardiovascular diseases, dental diseases, and fractures, have been conducted. These studies have taken advantage of the NDB’s strength as a highly comprehensive medical information database in Japan. Sampling Data, Accumulated Data, and Open Data can also be used effectively by taking advantage of their respective characteristics, and their use is expected to be promoted in the future.

Regarding limitations in using the NDB for research, in addition to the issues previously noted in review studies (including the lack of important information, absence of validation studies, and difficulties in making causal inferences with retrospective data ^(8)^), limitations, such as problems with patients not being included in the NDB, a lack of unique identifiers and a register of beneficiaries, as well as other operational issues, have been reported, and some strategies have been adopted to address them. Some of these limitations include those common to claims data studies, not just NDB (e.g., lack of information on confounders/covariates, outcomes, and other clinical content, limitations regarding patients not included in the claims database, and misclassification of data). Researchers need to consider the limitations of using the NDB in their studies and how these can be addressed. There was a wide variation in the amount of information provided on the NDB’s limitations among the studies included in this review, and insufficient details may lead readers to misunderstand the authors’ intent. An important future task would be to sort out the limitations associated with using the NDB for research purposes for both researchers and readers. In addition, it would be useful information for subsequent studies using NDB to clearly state the methods employed to address the NDB-specific limitations in each paper and to accumulate such findings.

The range of data available on the NDB has been expanding; the use of claims with fully publicly funded healthcare and linkage with long-term care insurance claims, as well as DPC data, are now possible. Methodologies to address the NDB’s limitations have also been developed, such as new IDs (ID5) that solve the problem of the patient’s identification number, where the same person cannot be tracked when insurance changes are present in recently provided data; moreover, other strategies, such as linking NDB data with mortality data from a municipality ^(27)^, are under consideration. Researchers using the NDB are expected to follow updated information and consider the limitations and available strategies in each study conducted.

### 4.1 Limitations of this study

Our review has several limitations. First, although the search strategies were determined by experienced information specialists and literature searches were conducted using three electronic sources (MEDLINE, EMBASE, and Ichushi-Web), our review might fail to include some NDB Open Data studies because Open Data studies are not included in the MHLW list used for the hand search. Second, the NDB data types were classified based on the descriptions in the text of each study. However, especially for General Data, the data type is often not identified; therefore, misclassification is possible. Third, our review does not consider the differences in the limitations depending on the time of year or research themes (e.g., research on pharmacoepidemiology and quality of care). The content and format of NDB data provided by the MHLW have changed over the years, and limitations related to the NDB and the strategies used to address them differ by the year and research theme of each study (e.g., laboratory data are not important for a research question on identifying prescription patterns and coverage of database is not important for a causal research question). It should be noted that this review was conducted in 2023. Finally, limitations not mentioned in each paper (e.g., inability to link household members) were not included because we used the descriptions in the included studies to extract the limitations of using the NDB for research purposes. Clearly stating the limitations of the NDB studies in the paper and reporting guidelines to support it may be necessary. Other methods, such as expert discussion, are required to create a comprehensive list of the limitations and strategies. These findings will help support future NDB research providing high-quality evidence.

## 5. Conclusions

The NDB has been used for various research purposes, including studies on the description of diseases and treatment patterns, examination of risk factors, the effectiveness of treatments and adverse events, quality of care, cost analysis, regional differences, and the impact of policy introduction, utilizing its strength as a comprehensive database of the health insurance claims information in Japan. The included studies note several limitations associated with using the NDB for research but also provide various strategies to address those limitations. In the future, NDB limitations and strategies will be organized across research fields. With these limitations identified and addressed, the NDB has the potential to provide useful information for researchers, healthcare providers, and policymakers, as well as contribute to the improvement of the healthcare policy in Japan.

## Article Information

### Conflicts of Interest

None

### Sources of Funding

This work was supported by the Research Project for the Establishment of an NDB Research System for Health Policy and Other Purposes through the 6NC Collaboration (2019-(1)-3).

### Acknowledgement

The authors thank Mr. Masahiko Watanabe and Ms. Chiemi Kataoka for developing and executing the search strategy.

### Author Contributions

This study was designed by MS, AI, TS, TK, MT, RT, MN, RK, KK, KI, NI, YI, KT, and HI. MS, AI, TS, TK, MT, RT, MN, RK, KI, NI, YI, and KT conducted the title, abstract, and full-text screening and data extraction for the included studies. MS, AI, TS, and KT drafted the initial manuscript. KK provided expert opinions. All authors reviewed and approved the final manuscript.

### Approval by Institutional Review Board (IRB)

Not applicable

## Supplement

Supplementary Files Supplementary File 1. Search strategy
Supplementary File 2. Study Selection Flowchart
Supplementary File 3. List of excluded studies with reasons
Supplementary File 4. Characteristics of included studies
Supplementary File 5. PRISMA-ScR Checklist
