# Supplementary material for: Literature Review of Studies Using the National Database of the Health Insurance Claims of Japan (NDB): Limitations and Strategies in Using the NDB for Research
Source: JMA J. 2023 Dec 27;7(1):10–20. doi: 10.31662/jmaj.2023-0078 (PMC10834238; doi:10.31662/jmaj.2023-0078)
Supplement: Supplementary Files — Supplementary File 1. Search strategy Supplementary File 2. Study Selection Flowchart Supplementary File 3. List of excluded studies with reasons Supplementary File 4. Characteristics of included studies Supplementary File 5. PRISMA-ScR Checklist [file 2433-3298-7-1-0010-s001.pdf]

Supplement file 1 Search strategy

Ichushi-Web	
#1	NDB/AL and (data/TA or information/TA or (database/TH or database/AL))
#2	(nation/TA or Japan/TA) and (health/TA or insurance/TA or administrative/TA) and claim/TA
#3	(nation/TA or Japan/TA) and claim/TA and database/TA
#4	national database/AL or national claims database/AL
#5	(claims data/TA or specific health checkups/TA or specific health guidance/TA) and (database/TH or database/AL)
#6	#1 or #2 or #3 or #4 or #5
#7	JMDC/AL
#8	Japan Medical Data Center/AL or (Japan/TA and medical/TA and data/TA and center/TA)
#9	employ/TA and health/TA and insurance/TA
#10	#7 or #8 or #9
#11	MDV/AL and (data/TA or information/TA or (database/TH or database/AL))
#12	Medical Data Vision/AL or Medical・Data・Vision/AL
#13	#11 or #12
#14	KDB/AL or kokuho database/AL or (Kokuho/TA and database/TA)
#15	((public health insurance/TH or public health insurance/AL) or health insurance/TA or medical insurance/TA or community insurance/TA) and (database/TH or database/AL)
#16	(Kokuho/TA or Shaho/TA or Kempo/TA or Kenpo/TA) and (data/TA orinformation/TA or (database/TH or database/AL))
#17	#14 or #15 or #16
#18	((insurance claim/TH or claim/AL) or health insurance claim/AL or insurance claim/AL or claim/AL or (health insurance/TH or health insurance/AL)) and (data/TA or information/TA or (database/TH or database/AL))
#19	health/TA and (insurance/TA or administrative/TA) and claim/TA
#20	#18 or #19
#21	(DPC/TA or PDPS/TA or DRG/TA) and (data/TA or information/TA or (database/TH or database/AL))
#22	(Diagnosis Procedure Combination／Per-Diem Payment System/TH or Per-Diem Payment System/AL or Diagnosis Procedure Combination/AL or Diagnosis Group/AL or Disease by Diagnosis/AL) and (data/TA or information/TA or (database/TH or database/AL))
#23	(diagnos/TA and procedure/TA and combination/TA) or (diagnos/TA and related/TA and groups/TA)
#24	#21 or #22 or #23
#25	medical/TA and electronic/TA and claim/TA
#26	#6 or #10 or #13 or #17 or #20 or #24 or #25
#27	(#26) and (PT=Article)
#28	(#26) and (PT=Review)
#29	(#26) and (PT=Commentary)
#30	(#26) and (PT=Conference Abstract)
	※Search was conducted in Japanese (translated into English for submission).
MEDLINE	
1	NDB.mp. and (Japan$.mp. or Japan.in.)
2	(nation$ adj5 insurance adj2 claim?).mp. and (Japan$.mp. or Japan.in.)
3	1 or 2
4	(JMDC or (employ$ adj2 health adj1 insurance)).mp. and (Japan$.mp. or Japan.in.)
5	(Japan adj1 medical adj1 data adj1 center).mp. or (Japan adj1 medical adj1 data adj1 center).in.
6	4 or 5
7	(MDV or (medical adj1 data adj1 vision)).mp. and (Japan$.mp. or Japan.in.)
8	(medical adj1 data adj1 vision).in.
9	7 or 8
10	(KDB.mp. and (Japan$.mp. or Japan.in.)) or (Kokuho adj1 database?).mp.
11	((DPC and database?) or PDPS).mp. and (Japan$.mp. or Japan.in.)
12	(diagnos$ adj1 procedure$ adj1 combination).mp. and (Japan$.mp. or Japan.in.)
13	(diagno$ adj1 related adj1 groups).mp. and (Japan$.mp. or Japan.in.)
14	(per-diem adj1 payment adj1 system).mp. and (Japan$.mp. or Japan.in.)
15	diagnosis-related groups.sh. and (Japan$.mp. or Japan.in.)
16	11 or 12 or 13 or 14 or 15
17	((medical or health or healthcare or administrative or insurance) adj5 claim?).mp. and (Japan$.mp. or Japan.in.)
18	(claim? adj1 (data or database)).mp. and (Japan$.mp. or Japan.in.)
19	(administrative claims, healthcare or insurance claim reporting or insurance claim review).sh. and (Japan$.mp. or Japan.in.)

20	(nationwide adj1 database adj5 Japan$).mp.
21	17 or 18 or 19 or 20
22	3 or 6 or 9 or 10 or 16 or 21

EMBASE	
#1	(ndb OR (nation* NEXT/5 insurance NEXT/2 claim*)) AND japan*
#2	(jmdc OR (japan NEXT/1 medical NEXT/1 data NEXT/1 center)) AND japan*
#3	(employ* NEXT/2 health NEXT/1 insurance) AND japan*
#4	#2 OR #3
#5	(mdv:ti,ab,kw OR (medical NEXT/1 data NEXT/1 vision)) AND japan*
#6	kdb AND japan* OR (kokuho NEXT/1 database*)
#7	(dpc AND database* OR pdps) AND japan*
#8	(diagnos* NEXT/1 procedure* NEXT/1 combination) AND japan*
#9	(diagnos* NEXT/1 related NEXT/1 group*) AND japan*
#10	'diagnosis related group'/exp AND japan*
#11	('per diem' NEXT/1 payment NEXT/1 system) AND japan*
#12	#7 OR #8 OR #9 OR #10 OR #11
#13	((medical OR health OR healthcare OR administrative OR insurance) NEXT/5 claim*) AND japan*
#14	(claim* NEAR/1 (data OR database*)) AND japan*
#15	nationwide NEXT/1 database* NEAR/5 japan*
#16	'health insurance'/exp AND claim* AND japan*
#17	'billing and claims'/exp AND japan*
#18	#13 OR #14 OR #15 OR #16 OR #17
#19	#1 OR #4 OR #5 OR #6 OR #12 OR #18
#20	#19 AND ('Article'/it OR 'Article in Press'/it)
#21	#19 AND 'review'/it
#22	#19 AND ('letter'/it OR 'note'/it OR 'short survey'/it)

Supplementary File 2 Study Selection Flowchart

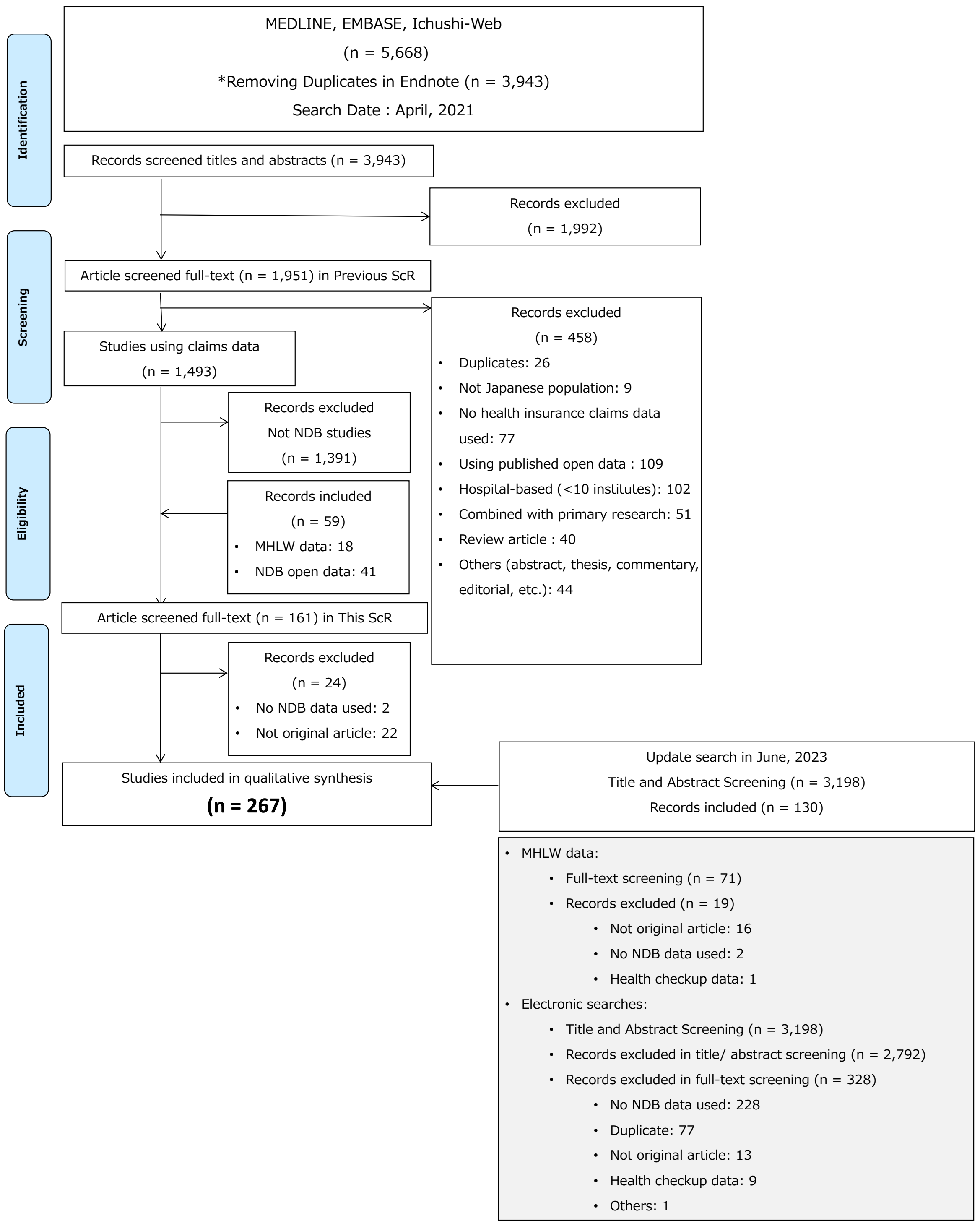

Supplementary File 3 List of excluded studies with reasons

ID (Author Year)	Title (*titles translated by author)	Reason
Yatsuhashi 2019	[Clinical Study of Liver Disease using National Database (NDB) : Trends in the Number of Patients with Chronic Hepatitis C and Hepatitis B]	Conference Report
Sugihara 2019	Regional clinical practice variation in urology: Usage example of the Open Data of the National Database of Health Insurance Claims and Specific Health Checkups of Japan	Urological Notes
Iwata 2021	[Survey of Dementia Drugs and PIM and Multi-Drug Combinations Using NDB Open Data]*	Conference Report
Miyake 2015	[Characteristics of heat-illness patients in Japan : Analysis from receipt data for the past 5 years]	Column
Iihara 2016	[Usage of Medication with Driving with Prohibition or Caution- Dose-limited drugs for the elderly should be used with caution]*	Review article
Ito 2014	[NDB Sampling Dataset: analysis and significance]*	Review article
Nakamura 2014	Abnormal behavior during influenza in Japan during the last seven seasons: 2006-2007 to 2012-2013	No NDB data used
Tsuneishi 2016	[National Database Analysis of the Relationship between the Number of Teeth and Medical Care Expenditures]*	Public relations article
Mitsutake 2015	[Effective use of National Database of Health Insurance Claims and Specific Health Checkups of Japan (NDB)]*	Review article
Okumura 2018	Antidementia drug use in Japan: bridging the research-to-practice gap	Letter to the editor
Okumura 2019	Prevalence, incidence, and persistence of ADHD drug use in Japan	Letter to the editor
Onishi 2020	[Report on "Research on the Appropriate Allocation of Medical Resources Based on the Road Transportation Network"]*	Research report
Ishibashi 2020	[A Study on Methods to Grasp Prices Reflecting Changes in the Quality of Medical Care: Examination of Estimation Methods and Trial Calculation Using Claims Data]*	Research Notes
Imai 2020	[A Study on Methods to Grasp Prices Reflecting Changes in the Quality of Medical Care: Statistical Measurement of the Quality of Medical Care Using Mortality Functions]*	Research Notes
Ruoyan Gai 2021	Estimates of inpatient costs for preterm and low-birthweight infants in Japan: An exploratory study using the National Database of Health Insurance Claims	Working Paper

Morimoto 2019	Actual practice of standard treatment for pulmonary nontuberculous mycobacteriosis in Japan	Short communication
Seki 2015	[Drug Utilization Study of Potential Drug-drug Interactions using the Sampling Dataset of the National Database of Health Insurance Claim Information]	Note
Suzuki 2021	Evaluation of Public Health Expenditure by Number of Teeth among Outpatients with Diabetes Mellitus	Short Communication
Kamata 2019	Status quo of osteoporosis treatment in Japan disclosed by the National Database of Health Insurance Claims and Specific Health Checkups: too late in treatment initiation and too few in treated patients?	Short Communication
Ohkusa 2018	Comparative study of preciseness in the regional variation of influenza in Japan among the National Official Sentinel Surveillance of Infectious Diseases and the National Database of Electronic Medical Claims	Brief Report
Sakai 2021	Decreased Administration of Life-Sustaining Treatment just before Death among Older Inpatients in Japan: A Time-Trend Analysis from 2012 through 2014 Based on a Nationally Representative Sample	Communication
Sugawara 2020	Association of moderately abnormal behavior and administered neuraminidase inhibitors	Brief Report
Iihara 2019	Decreased Risk of Fragility Fractures associated with Statin Use in the Older Japanese Population: a Nationwide Case–crossover Study	Report
Ueda 2020	[Responses to obstetric bleeding: Nationwide trend survey of obstetric critical bleeding using a database of information such as health insurance claims and specific health checkups]*	English paper available
Records excluded in update		
Sasayama 2021	Trends in Autism Spectrum Disorder Diagnoses in Japan, 2009 to 2019	Research Letter
Tsuneishil 2022	Use of the dental formula from the National Database of Health Insurance Claims and Specific Health Checkups of Japan	Review Article
Ishii 2022	[Health Impacts of Climate Change]*	Special Feature
Yamashita 2022	A Trial Study by Using Medical Receipt Data to Explore the Relationship between Agricultural activities and Health	Technical report
Tsukino 2019	[Current Treatment for Benign Prostatic Hyperplasia: The Multicenter Study of Miyazaki Urological-Network Group]*	No NDB data used

Tsukino 2020	[Patient Background Factors Affecting Prescription Drugs for Male Lower Urinary Tract Symptoms: The Multicenter Study of Miyazaki Urological-Network Group]	No NDB data used
Mori 2023	[Number of amblyopia diagnoses in FY2018 based on medical receipt data]	Conference Report
Sakai 2022	[Status of life-sustaining treatment before death in elderly patients: analysis using national data]*	Special Feature
Sasayama 2022	Trends in Diagnosed Attention- Deficit/Hyperactivity Disorder Among Children, Adolescents, and Adults in Japan From April 2010 to March 2020	Research Letter
Naito 2022	Delayed diagnosis of human immunodeficiency virus infection in people diagnosed with syphilis: A nationwide cohort study from 2011 to 2018 in Japan	Note
Ishida 2022	The 2018 Japan Floods Increased the Frequency of Yokukansan Prescriptions Among Elderly: A Retrospective Cohort Study	BRIEF RESEARCH REPORT
Noda 2022	Age-specific mortality associated with COVID-19 and seasonal influenza in Japan: using multiple population-based databases	Short report
Shida 2023	Use of National Database of Health Insurance Claims and Specific Health Checkups for examining practical utilization and safety signal of a drug to support regulatory assessment on postmarketing drug safety in Japan	Perspective
Tarasawa 2022	Medical resources and medical activities affect the rate of home death in cancer patients: Analysis by prefecture using Japanese National Database and public data	Preprints
Nishioka 2022	The Age of Death in Japanese patients with type 2 and type 1 diabetes: A descriptive epidemiological study	Short report
Kanaoka 2022	Reduction in Planned Percutaneous Coronary Interventions after the Policy Change for Ischemia Assessment in Japan	Letters
Nakao 2023	Impact of a national screening programme on obesity and cardiovascular risk factors	Health checkup data
Watanabe 2022	Factors Associated With Regional Differences in Healthcare Quality for Patients With Acute	Preprints
Itoshima 2022	Regional variations in primary percutaneous coronary intervention for acute myocardial infarction patients: A trajectory analysis using the national claims database in Japan	Preprints
Kanda 2022	Machine Learning Models Predicting Cardiovascular and Renal Outcomes and Mortality in Patients with Hyperkalemia	No NDB data used

Kanda 2022	Machine learning models for prediction of HF and CKD development in early-stage type 2 diabetes patients	No NDB data used
Ahmed 2022	Impact of Atrial Fibrillation on Outcomes of Aortic Valve Implantation	No NDB data used
Akada 2021	Real-world database analysis of the characteristics and treatment patterns of patients with endometrial cancer in Japan	No NDB data used
Akada 2022	Nationwide database analysis of insomnia, depression, and sleeping pill prescriptions in hepatocellular carcinoma patients	No NDB data used
Akada 2019	Database analysis of patients with hepatocellular carcinoma and treatment flow in early and advanced stages	No NDB data used
Akechi 2022	Risk of major depressive disorder in adolescent and young adult cancer patients in Japan	No NDB data used
Ando 2023	Seasonal exacerbation of rheumatoid arthritis detected by big claims data analysis: A retrospective population study	Duplicate
Arai 2023	A disproportionality analysis of the adverse effect profiles of methimazole and propylthiouracil in patients with hyperthyroidism using the Japanese Adverse Drug Event Report Database	No NDB data used
Arai 2022	Clinical characteristics and drug utilisation patterns in patients with chronic cough: a retrospective cohort study using a Japanese claims database	No NDB data used
Araki 2023	Relationship between the volume of cases and in-hospital mortality in patients with cardiogenic shock receiving short-term mechanical circulatory support	No NDB data used
Araki 2021	Prescription pattern analysis for antibiotics in working-age workers diagnosed with common cold	No NDB data used
Bouchi 2022	Retrospective nationwide study on the trends in first-line antidiabetic medication for patients with type 2 diabetes in Japan	Duplicate
Chen 2021	Characteristics of interstitial lung disease in patients from post-marketing data on metastatic breast cancer patients who received abemaciclib in Japan	No NDB data used
Deguchi 2021	Impact of Vonoprazan Triple-Drug Blister Packs on H. pylori Eradication Rates in Japan: Interrupted Time Series Analysis	No NDB data used
Den 2023	Epidemiology of Developmental Dysplasia of the Hip: Analysis of Japanese National Database	Duplicate

Endo 2022	Temporal trends in clinical characteristics and in-hospital mortality among patients with COVID-19 in Japan for waves 1, 2, and 3: A retrospective cohort study	No NDB data used
Endo 2022	Intensive care unit versus high-dependency care unit admission on mortality in patients with septic shock: a retrospective cohort study using Japanese claims data	No NDB data used
Fujioka 2022	Association between prenatal exposure to antidepressants and neonatal morbidity: An analysis of real-world data from a nationwide claims database in Japan	No NDB data used
Fujita 2023	Recent Trends in Treatment and Associated Costs of Primary Angle-Closure Glaucoma: A Retrospective Cohort Study	No NDB data used
Fujita 2023	Association between lifestyle habits and glaucoma incidence: a retrospective cohort study	No NDB data used
Fujita 2023	Impact of coronavirus disease 2019 pandemic on breast cancer surgery using the National Database of Japan	Duplicate
Fujita 2023	Hypnotics and injuries among older adults with Parkinson's disease: a nested case-control design	No NDB data used
Fujiwara 2021	Incidence of fractures among patients receiving medications for type 2 diabetes or chronic obstructive pulmonary disease and glucocorticoid users according to the National Claims Database in Japan	Duplicate
Fukunaga 2023	Real-world impact of dupilumab on asthma disease burden in Japan: The CROSSROAD study	No NDB data used
Goto 2021	Late-onset development of psoriasis in Japan: a population-based cohort study	No NDB data used
Goto 2022	Influence of pharmacists and infection control teams or antimicrobial stewardship teams on the safety and efficacy of vancomycin: A Japanese administrative claims database study	No NDB data used
Goto 2021	Association of subsequent treated shockable rhythm with outcomes after paediatric out-of-hospital cardiac arrests: A nationwide, population-based observational study	No NDB data used
Guan 2020	Treatment Patterns in Newly Diagnosed Multiple Myeloma Patients in Japan Using A Large-scale Claims Database: Retrospective Cohort Study	No NDB data used
Hagiwara 2021	A comparison of the safety and effectiveness of prasugrel and clopidogrel in younger population undergoing percutaneous coronary intervention: A retrospective study using a Japanese claims database	No NDB data used

Hattori 2022	Drug prescribing changes in the last year of life among homebound older adults: National retrospective cohort study	Duplicate
Hayashi 2022	Real-world evidence of the impact of obesity on residual teeth in the Japanese population: A cross-sectional study	No NDB data used
Higa 2022	Inverse Association Between Persistence With Antidepressant Medication and Onset of Chronic Pain in Patients With Depression: A Retrospective Cohort Study	No NDB data used
Higa 2023	Comparative effectiveness of early initiation of oral nonsteroidal anti-inflammatory drug and oral acetaminophen therapies on the time to knee replacement in patients with knee osteoarthritis in Japan	No NDB data used
Hiragi 2021	Association between the size of healthcare facilities and the intensity of hypertension therapy: a cross-sectional comparison of prescription data from insurance claims data	Duplicate
Hirose 2022	Association between nurse aide staffing and patient mortality after major cancer surgeries in acute care settings: A retrospective cohort study	No NDB data used
Hishimoto 2022	Treatment Discontinuation Among Patients with Schizophrenia Treated with Brexpiprazole and Other Oral Atypical Antipsychotics in Japan: A Retrospective Observational Study	No NDB data used
Honda 2023	Association Between Early Surgery and Postoperative Opioid Use in Patients With Lumbar Disc Herniation: A Propensity Score-Matching Analysis Using an Administrative Claims Database in Japan	No NDB data used
Honda 2020	Age in months and birth order in infant nonfatal injuries: A retrospective cohort study	No NDB data used
Horii 2019	Determination of factors affecting medication adherence in type 2 diabetes mellitus patients using a nationwide claim-based database in Japan	No NDB data used
Hoshino 2022	Direct health care cost of treatment and medication of biliary atresia patients using the National Database of Health Insurance Claims and Specific Health Checkups	Duplicate
Hozawa 2023	Real-world evaluation of asthma reliever therapy among continuous users of asthma maintenance medication in Japan: A retrospective cohort study using a claims database	No NDB data used
Huber 2020	Trends in micronutrient laboratory testing in switzerland: A 7-year retrospective analysis of healthcare claims data	No NDB data used

Ibayashi 2023	Estimation of the Number of Patients With Mitochondrial Diseases: A Descriptive Study Using a Nationwide Database in Japan	Duplicate
Igarashi 2022	Cost-Effectiveness Analysis of Omalizumab for Severe Allergic Asthma in Japan Using Real-World Evidence	No NDB data used
Ikeda 2022	Acute Pancreatitis in Japan: Comparison of before and after Revision of the Clinical Guidelines	No NDB data used
Iki 2022	Guideline adherence by physicians for management of glucocorticoid-induced osteoporosis in Japan: a nationwide health insurance claims database study	Duplicate
Iki 2022	Delayed initiation of anti-osteoporosis medications increases subsequent hip and vertebral fractures in patients on long-term glucocorticoid therapy: A nationwide health insurance claims database study in Japan	Duplicate
Iki 2023	Real-world effectiveness of anti-osteoporosis medications for the prevention of incident hip and clinical vertebral fractures in patients on long-term glucocorticoid therapy: A nationwide health insurance claims database study in Japan	Duplicate
Imada 2022	Impact of robot-assisted surgery appearance on reduction of annual blood transfusion cases in Japan: application of meta-analysis and NDB open data	Not original article
Imai 2020	Prescription of Colchicine with Other Dangerous Concomitant Medications: A Nation-Wide Survey Using the Japanese Claims Database	No NDB data used
Imai 2020	Association of the ward pharmacy service with active implementation of therapeutic drug monitoring for vancomycin and teicoplanin: an epidemiological surveillance study using Japanese large health insurance claims database	No NDB data used
Imai 2021	Probiotic prescription status of pediatric patients with otitis media receiving oral amoxicillin or amoxicillin/clavulanate from April 2016 to March 2017 using a Japanese health insurance claims database	No NDB data used
Imai 2021	A cross-sectional survey of hospitalization and blood tests implementation status in patients who received tolvaptan under 75 years of age using a Japanese claims database	No NDB data used
Imai 2021	Implementation Status of Liver Function Tests for Monitoring Benzbromarone-Induced Hepatotoxicity: An Epidemiological Survey Using the Japanese Claims Database	No NDB data used

Imaizumi 2022	Identifying high-risk population of depression: association between metabolic syndrome and depression using a health checkup and claims database	No NDB data used
Imura 2022	Epidemiological Study Regarding the Incidence of Venous Thromboembolism in Patients After Cancer Remission	No NDB data used
Inayama 2023	Real-world practice of estrogen therapy after surgery for endometrial cancer: a descriptive study using a Japanese claims database	No NDB data used
Inayama 2023	Hormone replacement therapy and cancer risks in perimenopausal women: A retrospective cohort study using a Japanese claims database	No NDB data used
Inose 2022	The intended purpose and regional patterns of use of antibiotics for managing Clostridioides (Clostridium) difficile infections: An analysis of the National Databese of Health Insurance Claims and Specific Health Checkups data of Japan	Not original article
Ishida 2021	The 2018 Japan Floods Increased the Frequency of Yokukansan Prescriptions Among Elderly: A Retrospective Cohort Study	Duplicate
Ishikawa 2023	Risk of major congenital malformations associated with first-trimester antihypertensives, including amlodipine and methyldopa: A large claims database study 2010-2019	No NDB data used
Ishikawa 2022	Risk of major congenital malformations associated with first-trimester exposure to propulsives: A health administrative database study in Japan	No NDB data used
Ishikura 2021	Leukotriene receptor antagonist use and dementia risk in patients with asthma: A retrospective cohort study	No NDB data used
Ishimaru 2018	Preoperative oral care and effect on postoperative complications after major cancer surgery	Duplicate
Ishizaki 2022	Tracheostomy decannulation rates in Japan: a retrospective cohort study using a claims database	No NDB data used
Isobe 2020	The number of overall hysterectomies per population with the perimenopausal status is increasing in Japan: A national representative cohort study	Duplicate
Itamoto 2022	No association between hospital volume and short-term outcomes of some common surgeries: a retrospective cohort study based on a Japanese nationwide database	No NDB data used
Ito 2022	A study of trends and factors associated with therapeutic drug monitoring (TDM) implementation for arbekacin treatment using a large Japanese medical claims database	No NDB data used

Itoshima 2021	The impact of the COVID-19 epidemic on hospital admissions for alcohol-related liver disease and pancreatitis in Japan	No NDB data used
Jamal 2021	Quantifying Regional and Health Care Variations to Identify Ways to Improve Hemodialysis Service Quality and Survival Outcomes	No NDB data used
Ji 2021	Trends and patterns in antibiotic prescribing for adult outpatients with acute upper respiratory tract infection in Japan, 2008-2018	No NDB data used
Jin 2022	Prescription trends in anti-seizure medications for adult patients with epilepsy in Japan: A retrospective cohort study using the database of health insurance claims between 2015 and 2019	No NDB data used
Jindai 2023	Decline in oral antimicrobial prescription in the outpatient setting after nationwide implementation of financial incentives and provider education: An interrupted time-series analysis	No NDB data used
Jingushi 2021	Low-intensity pulsed ultrasound is frequently used to treat fractures after osteosynthesis in elderly patients: A study using open data from the national database of health insurance claims of Japan	Duplicate
Kanaoka 2022	Trends and Factors Associated With Cardiac Rehabilitation Participation: Data From Japanese Nationwide Databases	Duplicate
Kanaoka 2023	Multifactorial effects of outpatient cardiac rehabilitation in patients with heart failure: a nationwide retrospective cohort study	Duplicate
Kanaoka 2023	Hospital- and Patient-Level Analysis of Quality Indicators in Acute Coronary Syndrome Care: A Nationwide Database Study	Duplicate
Kanaoka 2022	The impact of hospital case volume on the outcomes after catheter ablation for atrial fibrillation according to the ablation technology	Duplicate
Kanaoka 2021	Current Status and Effect of Outpatient Cardiac Rehabilitation After Percutaneous Coronary Intervention in Japan	Duplicate
Kanazawa 2023	Sex Differences in the Association Between Hypertension and Incident Atrial Fibrillation	No NDB data used
Kaneko 2021	Rates and risk factors for amputation in people with diabetes in Japan: a historical cohort study using a nationwide claims database	No NDB data used

Kaneko 2022	Impact of COVID-19 infection rates on admissions for ambulatory care sensitive conditions: nationwide difference-in-difference design in Japan	No NDB data used
Kashima 2022	The 2018 Japan Floods Increased Prescriptions of Antidementia Drugs Among Disaster Victims	Duplicate
Katano 2021	Trends in arthroplasty in Japan by a complete survey, 2014-2017	Duplicate
Kataoka 2023	Correlation between recurrence-free survival and overall survival after upfront surgery for resected colorectal liver metastases	No NDB data used
Kawabe 2022	Prevalence of therapeutic drug monitoring and adherence to imatinib in chronic myeloid leukemia in Japan	No NDB data used
Kawachi 2022	A Principal Component Analysis Approach to Estimate the Disability Status for Patients with Multiple Sclerosis Using Japanese Claims Data	No NDB data used
Kawai 2023	Patient characteristics, treatment patterns, and outcomes of hormone receptor-positive, human epidermal growth factor receptor 2-negative advanced breast cancer patients prescribed cyclin-dependent kinase 4 and 6 inhibitors: large-scale data analysis using a Japanese claims database	No NDB data used
Kawano 2021	Comprehensive Exploration of Medications That Affect the Bleeding Risk of Oral Anticoagulant Users	No NDB data used
Kawazoe 2022	Clinical characteristics of patients with polyarteritis nodosa based on a nationwide database in Japan	No NDB data used
Kikuchi 2022	【Current evidence and perspectives for hypertension management in Asia】Antihypertensive drug prescription trends for pregnant women with hypertension in acute hospitals in Japan	No NDB data used
Kikuchi 2022	Trends in the prescription of anti-seizure medicines for pregnant women outpatients with epilepsy during 2016–2020 in Japan	No NDB data used
Kim 2021	Variations in hip fracture inpatient care in Japan, Korea, and Taiwan: an analysis of health administrative data	No NDB data used
Kimura 2023	Impact of Potentially Inappropriate Medications on Kidney Function in Chronic Kidney Disease: Retrospective Cohort Study	No NDB data used

Kimura 2021	Real-world benefits of biologics for asthma: Exacerbation events and systemic corticosteroid use	No NDB data used
Kinugawa 2023	Impact of tolvaptan add-on treatment on patients with heart failure requiring long-term congestion management: A retrospective cohort study using a medical claim database in Japan	No NDB data used
Kobayashi 2023	Combined effects of blood pressure and glycemic status on risk of heart failure: a population-based study	No NDB data used
Kobayashi 2022	Survey of Anaphylaxis during Rasburicase Re-Administration in Patients with Hematological Malignancies Using a Japanese Claims Database	No NDB data used
Kohsaka 2022	Risk-Benefit Balance of Renin-Angiotensin-Aldosterone Inhibitor Cessation in Heart Failure Patients with Hyperkalemia	No NDB data used
Koizumi 2023	Effects of population age structure on parenteral antimicrobial use estimations	Duplicate
Komatsu 2022	Treatment Pattern for Advanced Gastric Cancer in Japan and Factors Associated with Sequential Treatment: A Retrospective Administrative Claims Database Study	No NDB data used
Komeda 2021	Comparison of Hospitalization Incidence in Influenza Outpatients Treated with Baloxavir Marboxil or Neuraminidase Inhibitors: A Health Insurance Claims Database Study	No NDB data used
Komeda 2021	Comparison of Household Transmission of Influenza Virus from Index Patients Treated with Baloxavir Marboxil or Neuraminidase Inhibitors: A Health Insurance Claims Database Study	No NDB data used
Kondo 2023	Prognosis in Patients With Cardiogenic Shock Who Received Temporary Mechanical Circulatory Support	No NDB data used
Konish 2023	Comparison of bleeding following gastrointestinal endoscopic biopsy in patients treated with and without direct oral anticoagulants	No NDB data used
Konishi 2022	Association between body mass index and incidence of breast cancer in premenopausal women: a Japanese nationwide database study	No NDB data used
Konishi 2022	Association of Operative Day of the Week with the Length of Stay and Total Hospitalization Costs in Patients with Partial Mastectomy: A Nationwide Database Study in Japan	No NDB data used
Konishi 2022	Short-Term Outcomes Following Breast Cancer Surgery With and Without Neoadjuvant Chemotherapy: A Nationwide Administrative Database Study in Japan	No NDB data used

Konishi 2023	Impact of the National Action Plan for Antimicrobial Resistance on Antibiotic Use for Mastitis Using a Japanese Nationwide Database	No NDB data used
Konishi 2022	Risk Factors for Postoperative Bleeding Following Breast Cancer Surgery: A Nationwide Database Study of 477,108 Cases in Japan	No NDB data used
Konishi 2022	Surgical and obstetric outcomes of breast cancer surgery during pregnancy: a nationwide database study in Japan	No NDB data used
Konishi 2023	Long-Term Risk of Being Bedridden in Elderly Patients Who Underwent Oncologic Surgery: A Retrospective Study Using a Japanese Claims Database	No NDB data used
Konishi 2023	Risk factors for arm lymphedema following breast cancer surgery: a Japanese nationwide database study of 84,022 patients	No NDB data used
Konuma 2022	Reducing Mortality of Single-Unit Unrelated Cord Blood Transplantation for Relapsed Acute Myeloid Leukemia after a Previous Allogeneic Transplantation: A Real-World Retrospective Study Over the Past 19 Years in Japan	No NDB data used
Koto 2021	Real-world treatment of gout and asymptomatic hyperuricemia: A cross-sectional study of Japanese health insurance claims data	No NDB data used
Koto 2023	Multimorbidity, Polypharmacy, Severe Hypoglycemia, and Glycemic Control in Patients Using Glucose-Lowering Drugs for Type 2 Diabetes: A Retrospective Cohort Study Using Health Insurance Claims in Japan	No NDB data used
Koto 2022	Temporal trends in the prevalence and characteristics of hypouricaemia: a descriptive study of medical check-up and administrative claims data	No NDB data used
Kubo 2022	Tracing all patients who received insured dialysis treatment in Japan and the present situation of their number of deaths	Duplicate
Kubota 2022	Effectiveness and Safety of Reduced and Standard Daily Doses of Direct Oral Anticoagulants in Patients with Nonvalvular Atrial Fibrillation: A Cohort Study Using National Database Representing the Japanese Population	Duplicate
Kunitomi 2021	Intergenerational comparison of 5-HT3RA in the prevention of chemotherapy-induced nausea and vomiting in gastric cancer patients receiving cisplatin-based chemotherapy: an observational study using a Japanese administrative claims database	No NDB data used

Kunitomi 2023	Efficacy of Daikenchuto in the prevention of bowel obstruction in patients with colorectal cancer undergoing laparoscopic surgery: An observational study using a Japanese administrative claims database	No NDB data used
Kuniyoshi 2022	Regional variation in the development of neonatal hyperbilirubinemia and relation with sunshine duration in Japan: an ecological study	Duplicate
Kurosaki 2020	Medical expenses for diabetes care in Japan: Analysis of inter-prefecture differences	Duplicate
Kusama 2021	Characteristics and limitations of national antimicrobial surveillance according to sales and claims data	Duplicate
Kuwana 2023	Cost-effectiveness analyses of biologic and targeted synthetic disease-modifying anti-rheumatic diseases in patients with rheumatoid arthritis: Three approaches with a cohort simulation and real-world data	No NDB data used
Kuwauchi 2022	Hemodynamic Monitoring Using a Pulmonary Artery Catheter Versus the Vigileo/FloTrac System during Elective Cardiac Surgery Based on Real-world Data in Japan	No NDB data used
Li 2021	Characterising the background incidence rates of adverse events of special interest for covid-19 vaccines in eight countries: multinational network cohort study	No NDB data used
Liu 2022	High-frequency HbA1c testing among older patients with diabetes in Japan: a longitudinal analysis using medical claims data	No NDB data used
Ma 2023	Prevalence trends of metabolic syndrome in residents of postdisaster Fukushima: a longitudinal analysis of Fukushima Health Database 2012-2019	Health checkup data
Machida 2023	Research note changes in the number of outpatient visits in Japan during the COVID-19 pandemic	Not original article
Machida 2022	Trends in drug prescriptions for type 2 diabetes, hypertension, and dyslipidemia among adults with non-alcoholic fatty liver disease	No NDB data used
Maeda 2021	Cesarean delivery rates for overall and multiple pregnancies in Japan: A descriptive study using nationwide health insurance claims data	Duplicate
Maeda 2022	Effect of Parenteral Energy or Amino Acid Doses on In-Hospital Mortality Among Patients With Aspiration Pneumonia: A Cohort Medical Claims Database Study	No NDB data used

Maeda 2021	Regional Disparity of Reperfusion Therapy for Acute Ischemic Stroke in Japan: A Retrospective Analysis of Nationwide Claims Data from 2010 to 2015	Duplicate
Maeda 2021	Nationwide temporal trend analysis of reperfusion therapy utilization and mortality in acute ischemic stroke patients in Japan	Duplicate
Maeda 2022	Association of comorbidities and medications with risk of asthma exacerbation in pediatric patients: a retrospective study using Japanese claims data	No NDB data used
Maeda-Minami 2023	Association between Statins and Incidence of Cancer in Patients with Dyslipidemia Using Large-Scale Health Insurance Claims Data	No NDB data used
Masaki 2023	Multikinase Inhibitor Treatment Patterns for Advanced Thyroid Cancer in Japan: An Administrative Claims Database Study	No NDB data used
Matsubayashi 2020	Prevalence, incidence, comorbidities, and treatment patterns among Japanese patients with acromegaly: a descriptive study using a nationwide claims database	Duplicate
Matsubayashi 2022	Impact of metabolic syndrome and metabolic dysfunction-associated fatty liver disease on cardiovascular risk by the presence or absence of type 2 diabetes and according to sex	No NDB data used
Matsuda 2021	Incorporating Unstructured Patient Narratives and Health Insurance Claims Data in Pharmacovigilance: Natural Language Processing Analysis of Patient-Generated Texts About Systemic Lupus Erythematosus	No NDB data used
Matsumura 2023	Proportion of subsequent clinic visits among persons without regular clinic visits who were screened as having hyperglycemia: A retrospective cohort study	No NDB data used
Matsuzaki 2023	Prognosis and incidence of infections in chronic kidney disease patients with membranous nephropathy enrolled in a large Japanese clinical claims database	No NDB data used
Mimura 2023	Prescribed daily-dose-based metrics of oral antibiotic use for hospitalized children in Japan	No NDB data used
Mita 2021	An alternative index for evaluating AMU and anti-methicillin-resistant Staphylococcus aureus agent use: A study based on the National Database of Health Insurance Claims and Specific Health Checkups data of Japan	Duplicate
Miyamoto 2023	Real-world comparison of in-hospital complications after catheter ablation for atrial fibrillation between non-antivitamin K anticoagulants and warfarin: A propensity-matched analysis using nation-wide database	No NDB data used

Miyamoto 2023	Evaluation of Plasmapheresis vs Immunoglobulin as First Treatment after Ineffective Systemic Corticosteroid Therapy for Patients with Stevens-Johnson Syndrome and Toxic Epidermal Necrolysis	No NDB data used
Miyashita 2023	Changes in the characteristics and outcomes of COVID-19 patients from the early pandemic to the delta variant epidemic: a nationwide population-based study	Duplicate
Miyazaki 2021	Evaluation of economic burden with biologic treatments in Crohn's disease patients: A mirror image study using an insurance database in Japan	No NDB data used
Mizuno 2021	Differences in aggressive treatments during the actively dying phase in patients with cancer and heart disease: an exploratory study using the sampling dataset of the National Database of Health Insurance Claims	Duplicate
Mizuno 2022	Search for Indexes to Evaluate Trends in Antibiotic Use in the Sub-Prefectural Regions Using the National Database of Health Insurance Claims and Specific Health Checkups of Japan	Duplicate
Mizuno 2022	Risk Factors for Recurrence of Peritonsillar Abscess	No NDB data used
Mizuta 2022	Effect of Postoperative Pain Management after Robot-Assisted Radical Prostatectomy: A Study on Reducing Hospital Length of Stay and Medical Costs Using Japanese Nationwide Database	No NDB data used
Mohri 2023	Risk of Lactic Acidosis in Hospitalized Diabetic Patients Prescribed Biguanides in Japan: A Retrospective Total-Population Cohort Study	Duplicate
Mori 2022	Medical expenditures for fragility hip fracture in Japan: a study using the nationwide health insurance claims database	Duplicate
Moribe 2023	Real-world treatment patterns of novel drugs in relapsed or refractory acute lymphoblastic leukemia patients in Japan	No NDB data used
Morimoto 2022	Cost-Effectiveness of Nab-Paclitaxel and Gemcitabine Versus Gemcitabine Monotherapy for Patients with Unresectable Metastatic Pancreatic Cancer in Japan	No NDB data used
Moriwaki 2021	Economic Evaluation of First-Line Pertuzumab Therapy in Patients with HER2-Positive Metastatic Breast Cancer in Japan	No NDB data used

Moroi 2023	Effectiveness and Safety of Reduced and Standard Daily Doses of Direct Oral Anticoagulants in Patients<br>with Nonvalvular Atrial Fibrillation: A Cohort Study Using National Database Representing the Japanese<br>Population	No NDB data used
Mukai 2020	Trends Associated with Hemorrhoids in Japan: Data Mining of Medical Information Datasets and the National Database of Health Insurance Claims and Specific Health Checkups of Japan(NDB) Open Data Japan	Duplicate
Muro 2023	Utility of Blood Culture in Patients with Community-Acquired Pneumonia: A Propensity Score-Matched Analysis Based on a Japanese National Health Insurance Database	Duplicate
Murota 2021	Cost-of-illness study for axillary hyperhidrosis in Japan	No NDB data used
Myojin 2022	Development of a New Method to Trace Patient Data Using the National Database in Japan	Duplicate
Nagai 2021	A Claims Database Analysis of Dose-Dependency of Metformin and Incidence of Lactic Acidosis in Japanese Patients with Type 2 Diabetes	No NDB data used
Nagai 2021	Effects of Increasing Metformin Dose vs Adding/Switching to Dipeptidyl Peptidase-4 Inhibitors on Glycemic Control in Patients with Type 2 Diabetes	No NDB data used
Nagakura 2021	The significant association between health examination results and population health: A cross-sectional ecological study using a nation-wide health checkup database in Japan	Health checkup data
Nagano 2023	Impact of the cefazolin shortage on the selection and cost of parenteral antibiotics during the supply disruption period in Japan: A controlled interrupted time series analysis	No NDB data used
Nagayama 2021	Medical Costs and Readmissions After Intensive Poststroke Rehabilitation: Japanese Claims Data	No NDB data used
Naito 2022	Delayed diagnosis of human immunodeficiency virus infection in people diagnosed with syphilis: A nationwide cohort study from 2011 to 2018 in Japan	Duplicate
Naito 2022	Analysis of antiretroviral therapy switch rate and switching pattern for people living with HIV from a national database in Japan	Duplicate
Naito 2022	Comorbidities and co-medications among 28 089 people living with HIV: A nationwide cohort study from 2009 to 2019 in Japan	Duplicate

Nakai 2022	Age-dependent association of discharge heart-failure medications with clinical outcomes in a super-aged society	Duplicate
Nakai 2022	Contemporary use of SGLT2 inhibitors in heart failure patients with diabetes mellitus: a comparison of DPP4 inhibitors in a nationwide electric health database of the superaged society	Duplicate
Nakai 2023	Impact of seasonal variation on hospital admission and in-hospital mortality of acute cardiovascular diseases; a contemporary nationwide database study	No NDB data used
Nakajima 2022	Geographic variations in rheumatoid arthritis treatment in Japan: A nationwide retrospective study using the national database of health insurance claims and specific health checkups of Japan	Duplicate
Nakamura 2022	Clinical Criteria for Persistent Inflammation, Immunosuppression, and Catabolism Syndrome: An Exploratory Analysis of Optimal Cut-Off Values for Biomarkers	No NDB data used
Nakamura 2023	Trends in surgical treatment for prostate cancer: Analysis of National Database Open Data in Japan	Not original article
Nakatoh 2021	Insufficient increase in bone mineral density testing rates and pharmacotherapy after hip and vertebral fracture: analysis of the National Database of Health Insurance Claims and Specific Health Checkups of Japan	Duplicate
Nakatoh 2021	Insufficient persistence to pharmacotherapy in Japanese patients with osteoporosis: an analysis of the National Database of Health Insurance Claims and Specific Health Checkups in Japan	Duplicate
Nakatoh 2021	Insufficient increase in bone mineral density testing rates and pharmacotherapy after hip and vertebral fracture: analysis of the National Database of Health Insurance Claims and Specific Health Checkups of Japan	Duplicate
Nakatoh 2022	Association of pharmacotherapy with the second hip fracture incidence in women: A retrospective analysis of the National Database of Health Insurance Claims and Specific Health Checkups of Japan	Duplicate
Neshige 2023	Are patients with Parkinson's disease at a lower risk of catching the common cold? Propensity score matching	No NDB data used

Nishioka 2022	The age of death in Japanese patients with type 2 and type 1 diabetes: A descriptive epidemiological study	Duplicate
Nishioka 2020	Incidence and seasonality of type 1 diabetes: a population-based 3-year cohort study using the National Database in Japan	Duplicate
Nishioka 2021	Association between influenza and the incidence rate of new-onset type 1 diabetes in Japan	Duplicate
Noda 2022	Age-specific Mortality Associated with COVID-19 and Seasonal Influenza in Japan: Using Multiple Population-based Databases	Duplicate
Nozawa 2022	Association Between HbA1c Levels and Diabetic Peripheral Neuropathy: A Case-Control Study of Patients with Type 2 Diabetes Using Claims Data	No NDB data used
Oami 2022	Temporal trends of medical cost and cost-effectiveness in sepsis patients: a Japanese nationwide medical claims database	No NDB data used
Oami 2023	Mortality analysis among sepsis patients in and out of intensive care units using the Japanese nationwide medical claims database: a study by the Japan Sepsis Alliance study group	No NDB data used
Oe 2021	Impact of prior cerebrovascular disease and glucose status on incident cerebrovascular disease in Japanese	No NDB data used
Ogawa 2023	ORIHIME study: real-world treatment patterns and clinical outcomes of 338 patients with acquired hemophilia A from a Japanese administrative database	No NDB data used
Ohara 2021	Fracture risk increased by concurrent use of central nervous system agents in older people: Nationwide case-crossover study	Duplicate
Okada 2023	A machine-learning-based prediction of non-home discharge among acute heart failure patients	No NDB data used
Okada 2022	Effects of medical service fee revision on reducing irrational psychotropic polypharmacy in Japan: an interrupted time-series analysis	No NDB data used
Okada 2023	Comparative effectiveness of long-acting injectable antipsychotics in patients with schizophrenia in Japan	No NDB data used
Okamoto 2022	Clinical Characteristics and Outcomes of Patients Presenting With Acute Myocardial Infarction Without Cardiogenic Shock	No NDB data used

Okazaki 2022	Impact of the 2018 Japan Floods on benzodiazepine use: a longitudinal analysis based on the National Database of Health Insurance Claims	Duplicate
Okazaki 2022	Impact of the 2018 Japan Floods on prescriptions for migraine: A longitudinal analysis using the National Database of Health Insurance Claims	Duplicate
Okazaki 2022	Increased prescriptions for irritable bowel syndrome after the 2018 Japan Floods: a longitudinal analysis based on the Japanese National Database of Health Insurance Claims and Specific Health Checkups	Duplicate
Onishi 2023	Development and usability of a hospital standardized ADL ratio (HSAR) for elderly patients with cerebral infarction: a retrospective observational study using administrative claim data from 2012 to 2019 in Japan	No NDB data used
Onishi 2022	Hospital-level characteristics of the standardised mortality ratio for ischemic heart disease: a retrospective observational study using Japanese administrative claim data from 2012 to 2019	No NDB data used
Onishi 2022	Evaluating the Hospital Standardized Home-Transition Ratios for Cerebral Infarction in Japan: A Retrospective Observational Study from 2016 through 2020	No NDB data used
Ono 2023	Risk of post-extraction bleeding with direct oral anticoagulant compared with warfarin: Retrospective cohort study using large scale claims data in Japan	No NDB data used
Osawa 2021	Physician visits and medication prescriptions for major chronic diseases during the COVID-19 pandemic in Japan: retrospective cohort study	No NDB data used
Osawa 2023	Association of the estimated glomerular filtration rate (eGFR) and/or proteinuria to predict the risk of initiation of dialysis in people with and without diabetes	No NDB data used
Ota 2021	Association between potassium supplementation and the occurrence of acute kidney injury in patients with hypokalemia administered liposomal amphotericin B: a nationwide observational study	No NDB data used
Otaka 2022	Influence of inpatient rehabilitation after crisis in patients with myasthenia gravis: A retrospective cohort study using a nationwide administrative database in Japan	No NDB data used
Oto 2021	Transdermal Fentanyl Usage in Working-age Patients Undergoing Cancer Treatment: Prescription Pattern Analysis Using Large Claims Data in Japan	No NDB data used

Ruzicka 2022	Characteristics of 2-drug regimen users living with HIV-1 in a real-world setting: A large-scale medical claim database analysis in Japan	No NDB data used
Sada 2022	The prevalence, burden of disease, and healthcare utilization of patients with eosinophilic granulomatosis with polyangiitis in Japan: a retrospective, descriptive cohort claims database study	No NDB data used
Saito 2022	Utilization of Dental Care and the Incidence of Dementia: A Longitudinal Study of an Older Japanese Cohort	No NDB data used
Saito 2023	Impact of sodium-glucose cotransporter-2 inhibitors on the risk of hip fracture in older patients in Japan using a nationwide administrative claims database: A matched case–control study	No NDB data used
Saito 2023	Prevalence of multimorbidity and its associations with hospitalisation or death in Japan 2014-2019: a retrospective cohort study using nationwide medical claims data in the middle-aged generation	No NDB data used
Sakai 2022	Unincreased risk of hospitalized infection under targeted therapies versus methotrexate in elderly patients with rheumatoid arthritis: a retrospective cohort study	No NDB data used
Sako 2021	Hospitalization for urinary tract infections in Japan, 2010-2015: a retrospective study using a national inpatient database	No NDB data used
Salinas 2023	Evaluation of VTE, MACE, and Serious Infections Among Patients with RA Treated with Baricitinib Compared to TNFi: A Multi-Database Study of Patients in Routine Care Using Disease Registries and Claims Databases	No NDB data used
Satake 2022	Real-World Data Analysis of Second-Line Antiangiogenic Targeted Treatments Following Anti-Epidermal Growth Factor Receptor Monoclonal Antibodies and First-Line FOLFOX for Patients with Metastatic Colorectal Cancer	No NDB data used
Sato 2021	Disproportionality by sex in the prescription of drugs capable of inducing parkinsonism for the elderly: A survey using statistics of Japanese national health claims from 2014 to 2017	Duplicate
Sato 2022	Trends in prophylactic antibiotic use for tooth extraction from 2015 to 2018 in Japan: An analysis using a health insurance claims database	No NDB data used

Satoh 2022	Actual impact of angiotensin II receptor blocker or calcium channel blocker monotherapy on renal function in real-world patients	No NDB data used
Sawada 2023	Cardiovascular risk of urate-lowering drugs: A study using the National Database of Health Insurance Claims and Specific Health Checkups of Japan	Duplicate
Sawaki 2023	Real-world treatment patterns of subsequent therapy after palbociclib in patients with advanced breast cancer in Japan	No NDB data used
Sawaki 2022	Real-world treatment patterns of palbociclib and blood count monitoring in patients with advanced breast cancer in Japan	No NDB data used
Shida 2023	Use of National Database of Health Insurance Claims and Specific Health Checkups for examining practical utilization and safety signal of a drug to support regulatory assessment on postmarketing drug safety in Japan	Duplicate
Shiga 2022	Long-term effectiveness of ustekinumab comparable to antitumor necrosis factor agents in patients with Crohn's disease	No NDB data used
Shimizu 2022	Hospital Frailty Risk Score predicts adverse events in older patients with vertebral compression fractures: Analysis of data in a nationwide in-patient database in Japan	No NDB data used
Shimizu 2022	Hospital Frailty Risk Score predicts adverse events in older patients with hip fractures after surgery: Analysis of a nationwide inpatient database in Japan	No NDB data used
Shimizu 2022	Diagnosis and treatment of influenza based on health insurance claims between the 2010-2011 and 2019-2020 influenza seasons in Japan	No NDB data used
Shimodaira 2021	Clinical course of ulcerative colitis associated with an age at diagnosis: A recent japanese database survey	No NDB data used
Shimodaira 2022	The risk of antibiotics and enterocolitis for the development of inflammatory bowel disease: a Japanese administrative database analysis	No NDB data used
Shimokawa 2022	Current status of pediatric sedation for MRI examinations in Japan: the National Database Open Data and random Sampling Data analysis of outpatient clinics	Duplicate
Shinjo 2021	Consultation-liaison psychiatry in Japan: a nationwide retrospective observational study	No NDB data used
Shinkawa 2021	Risk factors for venous thromboembolism in patients with nephrotic syndrome: a retrospective cohort study	No NDB data used

Sugimoto 2022	Trends in the prevalence and progression of diabetic retinopathy associated with hyperglycemic disorders during pregnancy in japan	No NDB data used
Sugiyama 2022	Liver biopsy implementation rate for diagnosis of NASH in Japan analysis of big data of health insurance claims	No NDB data used
Sugiyama 2022	Anisakiasis Annual Incidence and Causative Species, Japan, 2018-2019	No NDB data used
Sun 2023	Association between types of home healthcare and emergency house calls, hospitalization, and end-of-life care in Japan	Duplicate
Sun 2023	The Effect of Home Care Support Clinics on Hospital Readmission in Heart Failure Patients in Japan	Duplicate
Suzuki 2023	No association between major congenital malformations and exposure to Kampo medicines containing rhubarb rhizome: A Japanese database study	No NDB data used
Suzuki 2022	Dose-dependent relationship of blood pressure and glycaemic status with risk of aortic dissection and aneurysm	No NDB data used
Tajima 2021	Real-world anticancer medications for reproductive-age women with breast cancer by using a claims database in Japan	No NDB data used
Takabayashi 2022	Trend in prescription and treatment retention of molecular-targeted drugs in 121,131 Japanese patients with rheumatoid arthritis: A population-based real-world study	Duplicate
Takabayashi 2022	Incidence of opportunistic infections in patients with rheumatoid arthritis treated with different molecular-targeted drugs: A population-based retrospective cohort study	Duplicate
Takagi 2022	Dose-Dependent Effects of Amino Acids on Clinical Outcomes in Adult Medical Inpatients Receiving Only Parenteral Nutrition: A Retrospective Cohort Study Using a Japanese Medical Claims Database	No NDB data used
Takahashi 2022	Short- versus long-course antibiotic therapy for sepsis: a post hoc analysis of the nationwide cohort study	No NDB data used
Takahashi 2022	Trends in strong opioid prescription for cancer patients in Japan from 2010 to 2019: An analysis with large medical claims data	No NDB data used
Takahashi 2023	Real-world study of next-generation sequencing diagnostic biomarker testing for patients with lung cancer in Japan	No NDB data used

Takeshima 2023	Effects of Japanese policies and novel hypnotics on long-term prescriptions of hypnotics	No NDB data used
Takeuchi 2021	Sodium-glucose cotransporter-2 inhibitors and the risk of urinary tract infection among diabetic patients in Japan: Target trial emulation using a nationwide administrative claims database	Duplicate
Takeuchi 2021	Non-parametric approach for frequentist multiple imputation in survival analysis with missing covariates	No NDB data used
Takeyama 2021	Association of diagnostic delay with medical cost for patients with Crohn's disease: A Japanese claims-based cohort study	No NDB data used
Takura 2021	Development of a predictive model for integrated medical and long-term care resource consumption based on health behaviour: application of healthcare big data of patients with circulatory diseases	No NDB data used
Tamaki 2023	Hip and vertebral fracture risk after initiating antidiabetic drugs in Japanese elderly: a nationwide study	Duplicate
Tang 2023	Budget impact analysis of comprehensive genomic profiling for untreated advanced or recurrent solid cancers in Japan	No NDB data used
Tangri 2023	Prevalence of undiagnosed stage 3 chronic kidney disease in France, Germany, Italy, Japan and the USA: results from the multinational observational REVEAL-CKD study	No NDB data used
Terasaka 2023	Estimating the prevalence, clinical characteristics, and treatment patterns of hypertrophic cardiomyopathy in Japan: A nationwide medical claims database study	No NDB data used
Terashima 2021	Treatment patterns and medical costs after hepatectomy in real-world practice for patients with hepatocellular carcinoma in Japan	No NDB data used
Terashima 2022	Comparative analysis of medical costs after hepatectomy versus radiofrequency ablation in patients with hepatocellular carcinoma in real-world clinical practice	No NDB data used
Tokito 2023	Impact of Administering Intravenous Azithromycin within 7 Days of Hospitalization for Influenza Virus Pneumonia: A Propensity Score Analysis Using a Nationwide Administrative Database	No NDB data used
Tokunaga 2021	Cost analysis in helicobacter pylori eradication therapy based on a database of health insurance claims in japan	No NDB data used

Tomida 2023	Statin Persistence and Adherence among Older Initiators: A Nationwide Cohort Study Using the National Health Insurance Claims Database in Japan	Duplicate
Tonegawa-Kuji 2023	Impact of Low Body Mass Index on Cardiac Tamponade During Catheter Ablation for Atrial Fibrillation	No NDB data used
Tsuboi 2023	Differences in Parkinson's disease treatment between neurology and other departments in Japan	No NDB data used
Tsuchida 2022	Large-scale health insurance study showed that antibiotic use in infancy was associated with an increase in atopic dermatitis	No NDB data used
Tsuji 2022	Trends and patterns in the practice of pediatric sedation for magnetic resonance imaging in Japan: A longitudinal descriptive study from 2012 to 2019	No NDB data used
Tsuneishi 2021	Association between number of teeth and Alzheimer's disease using the National Database of Health Insurance Claims and Specific Health Checkups of Japan	Duplicate
Tsutsué 2021	Economic burden in treated Japanese patients with relapsed/refractory large B-cell lymphoma	No NDB data used
Tsutsué 2022	Cost drivers associated with diffuse large B-cell lymphoma (DLBCL) in Japan: A structural equation model (SEM) analysis	No NDB data used
Tsutsue 2022	Real-world assessment of myelodysplastic syndrome: Japanese claims data analysis	No NDB data used
Tsutsui 2023	Cost-effectiveness analysis of empagliflozin in patients with heart failure with reduced ejection fraction in Japan based on the EMPEROR-Reduced trial	No NDB data used
Uchiyama 2022	Medical Costs Associated with Insomnia Treatment with Suvorexant Monotherapy in Japan: Results from a Retrospective Cohort Study Using a Large-Scale Claims Database	No NDB data used
Ueno 2021	The disease burden of mucormycosis in Japan: results from a systematic literature review and retrospective database study	No NDB data used
Uno 2023	Trends in tolvaptan prescription and the association between hypernatremia and aging in tolvaptan-treated patients in Japan: Real-world data mining using Japanese databases	No NDB data used
Wada 2022	Significance of Ningen Dock as Screening System Through Treatment Rate of Past Diseases	No NDB data used
Wada-Isoe 2023	Non-ergot dopamine agonist therapy for Parkinson's disease in Japan: A claims database analysis	No NDB data used

Wakabayashi 2023	An Attempt to Replicate Randomized Trials of Diabetes Treatments Using a Japanese Administrative Claims and Health Checkup Database: A Feasibility Study	No NDB data used
Wakabayashi 2023	Impact of "time zero" of Follow-Up Settings in a Comparative Effectiveness Study Using Real-World Data with a Non-user Comparator: Comparison of Six Different Settings	No NDB data used
Wakasugi 2023	Prefecture-specific prevalence of overweight/obesity is associated with regional variation in the incidence of treated ESKD in Japan	Health checkup data
Watanabe 2022	Identification of the Components of Proton Pump Inhibitors and Potassium-Competitive Acid Blocker That Lead to Cardiovascular Events in Working-Age Individuals: A 12-Month Retrospective Cohort Study Using a Large Claims Database	No NDB data used
Watanabe 2022	Long-term effectiveness of a disease management program to prevent diabetic nephropathy: a propensity score matching analysis using administrative data in Japan	No NDB data used
Watanabe 2021	MIHARI project, a preceding study of MID-NET, adverse event detection database of Ministry Health of Japan-Validation study of the signal detection of adverse events of drugs using export data from EMR and medical claim data	No NDB data used
Yagi 2021	Current status of oral anticoagulant adherence in Japanese patients with atrial fibrillation: A claims database analysis	No NDB data used
Yaguchi 2022	Impact of Medication Adherence and Glycemic Control on the Risk of Micro- and Macrovascular Diseases in Patients with Diabetes	No NDB data used
Yahaba 2021	Antibiotics for hospitalized children with community-acquired pneumonia in Japan: Analysis based on Japanese national database	Duplicate
Yamada 2021	Associations of Systolic Blood Pressure and Diastolic Blood Pressure With the Incidence of Coronary Artery Disease or Cerebrovascular Disease According to Glucose Status	No NDB data used
Yamaguchi 2022	Rehabilitation services and related health databases, Japan	Not original article
Yamaguchi 2021	First-line antibiotic prescription patterns for acute otitis media in children: A descriptive study using Japanese claims data (2014-2018)	No NDB data used
Yamamoto 2023	Early Detection of Adverse Drug Reaction Signals by Association Rule Mining Using Large-Scale Administrative Claims Data	No NDB data used
Yamana 2021	Treatment of latent tuberculosis infection in patients receiving biologic agents	No NDB data used

Yamana 2022	Maoto plus neuraminidase inhibitor versus neuraminidase inhibitor alone for reducing hospitalization in older adults with seasonal influenza	No NDB data used
Yasuda 2022	Nutritional management of ICU patients receiving mechanical ventilation: A retrospective cohort study using a medical claims database	No NDB data used
Yatsuhashi 2021	Real-world hospital mortality of liver cirrhosis inpatients in Japan: a large-scale cohort study using a medical claims database: Prognosis of liver cirrhosis	No NDB data used
Yokoyama 2021	Trends and safety of robot-assisted partial nephrectomy during the initial 2-year period after government approval in Japan: A nationwide database study from 2016 to 2018	No NDB data used
Yokoyama 2023	The utility of the self-controlled study design for pharmacoepidemiological studies without an active comparator medication using a medical information database: An application to assess the risk of varenicline on cardiovascular outcomes	No NDB data used
Yokoyama 2023	Utilization of Big Data with a Focus on Administrative Claims Database	Not original article
Yokoyama 2023	Persistence and Safety of Golimumab in Elderly Patients with Rheumatoid Arthritis and Renal Dysfunction in a Real-World Setting	No NDB data used
Yonekura 2022	Trend in neuraxial morphine use and postoperative analgesia after cesarean delivery in Japan from 2005 to 2020	No NDB data used
Yonekura 2022	Current Epidemiology of the General Anesthesia Practice for Cesarean Delivery Using a Nationwide Claims Database in Japan: A Descriptive Study	No NDB data used
Yoneyama 2022	Comparison of laparoscopic and open inguinal hernia repair in adults: A retrospective cohort study using a medical claims database	No NDB data used
Yoneyama 2023	Recent Trend of Using Computed Tomography to Diagnose Pediatric Appendicitis at the First Hospital Visit: A Descriptive Study Using a Medical Claims Database	No NDB data used
Yoshida 2022	Changes in Platelet Counts and Thrombocytopenia Risk in Patients with Chronic Liver Disease with Different Etiologies Using Real-World Japanese Data	No NDB data used
Yoshida 2023	Effects of 2018 Japan floods on healthcare costs and service utilization in Japan: a retrospective cohort study	Duplicate
Yoshii 2021	Association between allergic or autoimmune diseases and incidence of endometriosis: A nested case-control study using a health insurance claims database	No NDB data used

Yoshisue 2022	Clinical Characteristics, Health Care Resource Utilization, and Prescription Patterns of Japanese Patients with Physician-Diagnosed Allergic Rhinitis: A Secondary Use of Database Study	No NDB data used
Kato 2022	[Survey on BMI and lifestyle habits in Kanagawa Prefecture: Analysis based on specified health checkups]*	Health checkup data
Iwata 2021	[Survey on dementia drugs, potentially inappropriate medications (PIMs), and polypharmacy using open data from the National Database of Health Insurance Claims and Specific Health Checkups of Japan (NDB)]*	Not original article
Miyai 2021	[Implementation status of voriconazole therapeutic drug monitoring: analysis of data from a large Japanese health insurance claims database]	No NDB data used
Kondo 2021	[Effects of medical cost reduction for outpatient cardiac rehabilitation]	No NDB data used
Kaneshige 2022	[Study of factors related to mortality rate : Analysis using NDB open data]	Health checkup data
Sasaki 2022	[A Survey on Acne Conglobata Using a Health Insurance Claims Database]	No NDB data used
Sasaki 2023	[A Survey of Antibiotic Prescription and Treatment Continuation Rates in Acne Therapy Using a Health Insurance Claims Database]	No NDB data used
Deguchi 2022	[Relationship between smoking rate and major health indicators in NDB open data Japan : an ecological study comparing 47 prefectures and the status of Wakayama Prefecture]	Health checkup data
Mori 2023	[Number of amblyopia diagnoses in FY2018 based on medical receipt data]	Duplicate
Suga 2019	[Current status of geriatric neurosurgery in Japan: Analysis using NDB open data]*	Not original article
Nishioka 2022	[Trends in the estimated average length of hospital stays and changes in hospitalization trends due to COVID-19, as reported by IKA Medias]*	No NDB data used
Aono 2021	[Prevalences of coronary risk factors in Wakayama Prefecture : Comparison to the national level using NDB Open Data Japan]	Health checkup data
Ishimura 2023	[Proposal of a drug cost reduction method based on pharmacist judgment : A case of metformin hydrochloride tablets]	Not original article
Ishimura 2022	[Impact of Introduction of an Authorized Generic Drug Supply System on the Pharmaceutical Market Analyzed Using Statin Prescription Data]	Not original article

Maeda 2021	[Prescription of antiviral drugs for children with influenza in Japan : a study based on health insurance claims data]	No NDB data used
Maeda 2021	[Analysis of the Status of Reimbursement for Home Medical Care Management Guidance and Additional Reimbursement for Provision of Sanitary Materials Using the National Database of Health Insurance Claims and Specific Health Checkups of Japan]	Not original article
Aino 2021	[Characteristics of patients newly diagnosed with Alzheimer's disease and factors contributing to the onset]	Duplicate
Niwase 2022	[Proposal of Next-Generation Analytic Platform for the National Database of Health Insurance Claims and Specific Health Checkups of Japan (NDB) Using Parallel Distributed Processing]	Others
Tamura 2021	[Increasing Cases of Cataract Surgery with Intraocular Lens Suturing : a Study of All Cases Based on Open Data from the National Database of Health Insurance Claims and Specific Health Checkups of Japan (NDB)]	Not original article
Bouchi 2022	[Fact-finding survey on diabetes drugs initially administered for type 2 diabetes in Japan: Analysis using the large-scale database NDB]*	Not original article
Suenaga 2021	Factors related to life expectancy and healthy life expectancy in prefectures: An ecological study using the National Database	Health checkup data
Hamaguchi 2022	Study of factors related to healthy life expectancy using real world data	Health checkup data
Takeshita 2021	[Study on factors related to hospitalization period of acute cerebral infarction using NDB sampling data set]	Duplicate

Supplementary File 4 Characteristics of included studies

ID (Author Year)	Title (*titles translated by author)	Database	Research area/ theme	Research area/ theme-2	Disease	Disease-2	Notes on disease	Age	Notes on age	ID used for analysis
Aino 2021	[Characteristics of patients newly diagnosed with Alzheimer's disease and factors contributing to the onset]	General Data	Clinical epidemiology, course of diseases		Diseases of the nervous system		Alzheimer's disease	No age limit/ Others		
Akazawa 2018	Cost-Minimization Analysis of Deep-Brain Stimulation Using National Database of Japanese Health Insurance Claims	General Data	Health economics		Diseases of the nervous system		Parkinson's disease and other movement disorders	No age limit/ Others		
Ando 2023	Seasonal exacerbation of rheumatoid arthritis detected by big claims data analysis: A retrospective population study	General Data	Clinical epidemiology, course of diseases		Diseases of the musculoskeletal system and connective tissue		rheumatoid arthritis	No age limit/ Others		ID1 and ID2
Arakawa 2015	[Actual status of prescription patterns of anxiolytics and hypnotics in outpatients using National Database of Health Insurance Claim Information and Specific Medical Checkups]	Sampling data	Medical treatment status		Mental, Behavioral and Neurodevelopmental disorders	Diseases of the nervous system	anxiolytics, hypnotics	No age limit/ Others		ID1
Azuma 2022	Evaluation of the Correspondence between the Concentration of Antimicrobials Entering Sewage Treatment Plant Influent and the Predicted Concentration of Antimicrobials Using Annual Sales, Shipping, and Prescriptions Data	Open data	Research methodology		Others		antimicrobial concentrations	No age limit/ Others		
Bouchi 2021	Retrospective nationwide study on the trends in first-line antidiabetic medication for patients with type 2 diabetes in Japan	General Data	Medical treatment status	Health economics	Endocrine, nutritional, and metabolic diseases		Type 2 diabetes	Adults	those who were aged <20 years were excluded	ID1
Chun 2022	Economics of drug-coated balloons for arteriovenous fistula stenosis in Japan and Korea based on the IN.PACT AV access trial	Open data	Health economics	Socioeconomic comparison	Diseases of the circulatory system		arteriovenous fistula stenosis	No age limit/ Others		
Chwen Ni 2022	Comprehensive Assessment of the Universal Healthcare System in Dentistry Japan: A Retrospective Observational Study	Open data	Health economics		Diseases of the digestive system		dentistry	No age limit/ Others		
Den 2023	Epidemiology of developmental dysplasia of the hip: analysis of Japanese national database	General Data	Clinical epidemiology, course of diseases	Socioeconomic comparison	Congenital malformations, deformations and chromosomal abnormalities	Injury, poisoning and certain other consequences of external causes	developmental dysplasia of the hip, hip dislocation	Children	born between 2011 and 2013 and assigned DDH-dislocation-related disease codes during 2011–2018	ID1, ID2
Ehara 2017	[Current status of hospitalization of children by prefecture as estimated from NDB open data]*	Open data	Medical treatment status		Others		hospital admissions	Children	aged <15 years	
Fujita 2018	Hepatitis B virus reactivation in patients with rheumatoid arthritis: Analysis of the National Database of Japan	General Data	Clinical epidemiology, course of diseases	Quality of care	Certain infectious and parasitic diseases	Diseases of the musculoskeletal system and connective tissue	hepatitis B virus, rheumatoid arthritis	Adults	30-79 years	
Fujita 2023	Changes in the number of cancer diagnosis practices due to the COVID-19 pandemic: interrupted time-series analysis using the National Database of Japan	Sampling data	Medical treatment status	Others	Neoplasms	Certain infectious and parasitic diseases	five major cancers, COVID-19	No age limit/ Others		
Fujita 2023	Impact of coronavirus disease 2019 pandemic on breast cancer surgery using the National Database of Japan	Sampling data	Medical treatment status	Others	Neoplasms	Certain infectious and parasitic diseases	breast cancer, COVID-19	No age limit/ Others		
Fujita 2023	Changes in colorectal cancer treatment during the COVID-19 pandemic in Japan: Interrupted time-series analysis using the National Database of Japan	Sampling data	Medical treatment status	Others	Neoplasms	Certain infectious and parasitic diseases	colorectal cancer, COVID-19	No age limit/ Others		ID1

ID (Author Year)	Title (*titles translated by author)	Database	Research area/ theme	Research area/ theme-2	Disease	Disease-2	Notes on disease	Age	Notes on age	ID used for analysis
Fujiwara 2021	Incidence of fractures among patients receiving medications for type 2 diabetes or chronic obstructive pulmonary disease and glucocorticoid users according to the National Claims Database in Japan	Accumulated data	Clinical epidemiology, course of diseases		Injury, poisoning and certain other consequences of external causes	Diseases of the musculoskeletal system and connective tissue	fracture (hip fracture, radius fracture, clinical vertebral fractures)	Adults	aged >= 40 years	
Fukuda 2018	Inpatient Expenditures Attributable to Hospital-Onset Clostridium difficile Infection: A Nationwide Case-Control Study in Japan	General Data	Health economics		Certain infectious and parasitic diseases		Clostridium difficile infections	No age limit/ Others		
Fukuda 2019	[The Development of Dataset Tables for NDB Analyses]	General Data	Research methodology		Others		analytical dataset tables	No age limit/ Others		ID1 or ID2 matched
Fukuda 2019	[A comparison of correction methods for medical fee revisions in health expenditure analyses using claims data]	General Data	Research methodology		Others		correction methods for medical fee revisions	No age limit/ Others		ID1 or ID2 matched
Fukuda 2020	Differences in healthcare expenditure estimates according to statistical approach: A nationwide claims database study on patients with hepatocellular carcinoma	General Data	Research methodology	Health economics	Neoplasms		hepatocellular carcinoma	Adults	≥20 years of age	
Fukuda 2020	Healthcare resources attributable to methicillin-resistant Staphylococcus aureus orthopedic surgical site infections	General Data	Health economics		Injury, poisoning and certain other consequences of external causes		MRSA SSIs	No age limit/ Others		
Fukuda 2020	Healthcare Expenditures for the Treatment of Patients Infected with Hepatitis C Virus in Japan	General Data	Health economics		Certain infectious and parasitic diseases		Hepatitis C Virus	Adults	≥20 years of age	
Fukuda 2020	Comparing Retreatments and Expenditures in Flow Diversion Versus Coiling for Unruptured Intracranial Aneurysm Treatment: A Retrospective Cohort Study Using a Real-World National Database	General Data	Intervention effect	Health economics	Diseases of the circulatory system		unruptured intracranial aneurysms	No age limit/ Others		
Fukuma 2020	Quality of Care in Chronic Kidney Disease and Incidence of End-stage Renal Disease in Older Patients: A Cohort Study	General Data	Quality of care	Intervention effect	Diseases of the genitourinary system		chronic kidney disease, end-stage renal disease	Older persons	≥65 years of age	
Hagiwara 2015	The effectiveness of risk communication regarding drug safety information: a nationwide survey by the Japanese public health insurance claims data	General Data	Health policy evaluation and utilization		Certain infectious and parasitic diseases	Diseases of the musculoskeletal system and connective tissue	hepatitis, rheumatoid arthritis	No age limit/ Others		ID1
Hagiwara 2017	The Survey of the Compliance Situation to the Antihypertensive Therapy Guideline by Analyzing Japanese National Claims Data	Sampling data	Medical treatment status	Quality of care	Diseases of the genitourinary system		kidney disease	No age limit/ Others		ID1
Hasegawa 2022	Short-term associations of ambient air pollution with hospital admissions for ischemic stroke in 97 Japanese cities	General Data	Clinical epidemiology, course of diseases	Others	Diseases of the circulatory system		ischemic stroke	Adults	aged <20 years were excluded	ID1 and ID2
Hashimoto 2020	Indications and classes of outpatient antibiotic prescriptions in Japan: A descriptive study using the national database of electronic health insurance claims, 2012-2015	General Data	Medical treatment status		Others		outpatient antibiotic prescription	No age limit/ Others		ID1
Hashimoto 2022	Incidence of Sympathetic Ophthalmia after Inciting Events: A National Database Study in Japan	General Data	Clinical epidemiology, course of diseases		Diseases of the eye and adnexa		Sympathetic Ophthalmia	No age limit/ Others		2 types of identifiers
Hattori 2022	Drug prescribing changes in the last year of life among homebound older adults: national retrospective cohort study	General Data	Medical treatment status		Others		Drug prescribing changes, homebound older adults	Older persons	older adults aged ≥75 years	ID1
Hattori 2022	National trends in gastrostomy in older adults between 2014 and 2019 in Japan	Open data	Medical treatment status		Others		gastrostomy	Older persons	aged ≥65 years	

ID (Author Year)	Title (*titles translated by author)	Database	Research area/ theme	Research area/ theme-2	Disease	Disease-2	Notes on disease	Age	Notes on age	ID used for analysis
Hayashi 2018	Dissemination of cognitive behavioral therapy for mood disorder under the national health insurance scheme in Japan: A descriptive study using the National Database of Health Insurance Claims of Japan with special focus on Japan's southwest region	General Data	Medical treatment status		Mental, Behavioral and Neurodevelopmental disorders		mood disorder	No age limit/ Others		
Hayashi 2019	Data regarding fracture incidence according to fracture site, month, and age group obtained from the large public health insurance claim database in Japan	General Data	Clinical epidemiology, course of diseases	Research methodology	Injury, poisoning and certain other consequences of external causes		fracture	No age limit/ Others		ID0
Hayashi 2019	Variation in fracture risk by season and weather: A comprehensive analysis across age and fracture site using a National Database of Health Insurance Claims in Japan	General Data	Clinical epidemiology, course of diseases		Injury, poisoning and certain other consequences of external causes		fracture	No age limit/ Others		
Hayashi 2020	How was cognitive behavioural therapy for mood disorder implemented in Japan? A retrospective observational study using the nationwide claims database from FY2010 to FY2015	Accumulated data	Medical treatment status		Mental, Behavioral and Neurodevelopmental disorders		mood disorder	No age limit/ Others		
Higuchi 2022	[Clozapine Use in Japan Based on National Database of Health Insurance Claims and Specific Health Checkups Open Data: Disparities by Region, Age, and Sex]	Open data	Medical treatment status	Socioeconomic comparison	Mental, Behavioral and Neurodevelopmental disorders		clozapine use	No age limit/ Others		
Hiragi 2021	Association between the size of healthcare facilities and the intensity of hypertension therapy: a cross-sectional comparison of prescription data from insurance claims data	Sampling data	Medical treatment status	Health policy evaluation and utilization	Diseases of the circulatory system		hypertension	No age limit/ Others		
Hori 2022	Trends in outpatient rehabilitation practices in Japan: analysis using the National Database of Health Insurance Claims Open Data	Open data	Medical treatment status	Socioeconomic comparison	Others		outpatient rehabilitation	No age limit/ Others		
Hoshino 2022	Direct health care cost of treatment and medication of biliary atresia patients using the National Database of Health Insurance Claims and Specific Health Checkups.	General Data	Health economics		Congenital malformations, deformations and chromosomal abnormalities		biliary atresia	Children	ages 0 days to 4 years	
Hoshino 2022	Relationship between diverting stoma and adjuvant chemotherapy in patients with rectal cancer: a nationwide study using the National Database of Health Insurance Claims and Specific Health Checkups of Japan	General Data	Intervention effect		Neoplasms		rectal cancer	No age limit/ Others		
Hosoi 2023	Relationship between antidementia medication and fracture prevention in patients with Alzheimer's dementia using a nationwide health insurance claims database	General Data	Intervention effect		Diseases of the nervous system	Injury, poisoning and certain other consequences of external causes	Alzheimer's dementia, fracture	Older persons	aged ≥ 65 years	ID 1 and 2
Hosomi 2016	[Association of Antipsychotic Use with Extrapyramidal Symptoms: Data Mining of the Japanese National Insurance Claims Database]	General Data	Intervention effect		Mental, Behavioral and Neurodevelopmental disorders	Diseases of the nervous system	Extrapyramidal Symptoms (Antipsychotic Use)	No age limit/ Others		ID
Ibayashi 2021	Estimation of the number of patients with mitochondrial diseases: A descriptive study using a nationwide database in Japan	General Data	Clinical epidemiology, course of diseases		Endocrine, nutritional, and metabolic diseases		patients with mitochondrial diseases	No age limit/ Others		ID1
Igari 2020	A retrospective observational study of antimicrobial treatment for non-tuberculous mycobacteria disease using a nationwide claims database in Japan	Sampling data	Medical treatment status	Quality of care	Certain infectious and parasitic diseases		non-tuberculous mycobacteria	No age limit/ Others		
Igari 2020	A retrospective observational study of antibiotics treatment for sepsis using a nationwide claim database in Japan	Sampling data	Medical treatment status	Clinical epidemiology, course of diseases	Certain infectious and parasitic diseases		antibiotics treatment for sepsis	Adults	aged 15 years and older	

ID (Author Year)	Title (*titles translated by author)	Database	Research area/ theme	Research area/ theme-2	Disease	Disease-2	Notes on disease	Age	Notes on age	ID used for analysis
Igari 2020	Epidemiology and treatment outcome of pneumonia: Analysis based on Japan national database	Sampling data	Clinical epidemiology, course of diseases		Certain infectious and parasitic diseases	Diseases of the respiratory system	pneumonia	Adults	aged 15 years and older	
Ihana-Sugiyama 2023	Patient referral flow between physician and ophthalmologist visits for diabetic retinopathy screening among Japanese patients with diabetes: A retrospective cross-sectional cohort study using the National Database	General Data	Medical treatment status	Quality of care	Endocrine, nutritional, and metabolic diseases		diabetic retinopathy screening	No age limit/ Others		
Iihara 2014	[Survey of Usage of Medication with Driving with Prohibition or Caution by the National Health Insurance Claims Database in Japan]	Sampling data	Medical treatment status	Quality of care	Others		prohibitions or cautions on driving	Adults	25 years and older	ID1
Iihara 2016	Polypharmacy of medications and fall-related fractures in older people in Japan: a comparison between driving-prohibited and driving-cautioned medications	Sampling data	Clinical epidemiology, course of diseases	Medical treatment status	Injury, poisoning and certain other consequences of external causes		fall-related fractures	Older persons	aged ≥65 years	
Iihara 2019	Fragility Fractures in Older People in Japan Based on the National Health Insurance Claims Database	General Data	Clinical epidemiology, course of diseases		Diseases of the musculoskeletal system and connective tissue	Injury, poisoning and certain other consequences of external causes	fragility fracture	Older persons	65 years and older	ID1 and ID2
Ikeda 2023	Effect of the Diabetic Nephropathy Aggravation Prevention Program on medical visit behavior in individuals under the municipal national health insurance	General Data	Health policy evaluation and utilization		Endocrine, nutritional, and metabolic diseases		diabetic nephropathy	No age limit/ Others		id1n
Iki 2022	Guideline adherence by physicians for management of glucocorticoid-induced osteoporosis in Japan: a nationwide health insurance claims database study	General Data	Quality of care		Diseases of the musculoskeletal system and connective tissue		glucocorticoid-induced osteoporosis	Adults	aged ≥ 50 years	ID
Iki 2022	Delayed initiation of anti-osteoporosis medications increases subsequent hip and vertebral fratures in patients on long-term glucocorticoid therapy: A nationwide health insurance claims database study in Japan	General Data	Intervention effect	Medical treatment status	Injury, poisoning and certain other consequences of external causes	Diseases of the musculoskeletal system and connective tissue	hip and vertebral fracture, glucocorticoid-induced osteoporosis	Adults	aged ≥ 50 years	ID
Iki 2023	Real-world effectiveness of anti-osteoporosis medications for the prevention of incident hip and clinical vertebral fractures in patients on long-term glucocorticoid therapy: A nationwide health insurance claims database study in Japan	General Data	Intervention effect		Injury, poisoning and certain other consequences of external causes	Diseases of the musculoskeletal system and connective tissue	hip and vertebral fracture, glucocorticoid-induced osteoporosis	Adults	aged ≥50 years	ID
Imai 2022	Antiresorptive drugs and the risk of femoral shaft fracture in men and women with osteoporosis: A cohort study using the National Database of Health Insurance Claims of Japan	General Data	Intervention effect		Diseases of the musculoskeletal system and connective tissue	Injury, poisoning and certain other consequences of external causes	osteoporosis, femoral fracture	No age limit/ Others		ID1 and ID2
Inoue 2019	Regional variance in patterns of prescriptions for chronic kidney disease in Japan	Open data	Medical treatment status	Socioeconomic comparison	Diseases of the genitourinary system		chronic kidney disease	No age limit/ Others		
Inoue 2020	Regional Variance of the Early Use of Tolvaptan for Autosomal Dominant Polycystic Kidney Disease	Open data	Medical treatment status	Socioeconomic comparison	Congenital malformations, deformations and chromosomal abnormalities		autosomal dominant polycystic kidney disease	No age limit/ Others		
Inoue 2021	Regional distribution of cardiologists and prescription patterns of sodium-glucose transporter-2 inhibitors in Japan	Open data	Medical treatment status	Socioeconomic comparison	Endocrine, nutritional, and metabolic diseases		SGLT2	No age limit/ Others		
Ishida 2022	Nationwide Long-Term Evaluation of Polypharmacy Reduction Policies Focusing on Older Adults in Japan	Open data	Health policy evaluation and utilization		Others		polypharmacy	No age limit/ Others		

ID (Author Year)	Title (*titles translated by author)	Database	Research area/ theme	Research area/ theme-2	Disease	Disease-2	Notes on disease	Age	Notes on age	ID used for analysis
Ishida 2022	Effectiveness of polypharmacy reduction policy in Japan: nationwide retrospective observational study	Open data	Health policy evaluation and utilization		Others		polypharmacy	No age limit/ Others		
Ishimaru 2018	Preoperative oral care and effect on postoperative complications after major cancer surgery	General Data	Intervention effect		Neoplasms		patients who underwent surgery for head and neck, oesophageal, gastric, colorectal, lung or liver cancer	Adults	excluded aged less than 18 years	
Ishimaru 2022	Prevalence, Incidence Rate, and Risk Factors of Medication-Related Osteonecrosis of the Jaw in Patients With Osteoporosis and Cancer: A Nationwide Population-Based Study in Japan	General Data	Clinical epidemiology, course of diseases	Intervention effect	Diseases of the digestive system		Medication-related osteonecrosis of the jaw	No age limit/ Others		
Isobe 2020	Correlation between the number of laparoscopy-qualified gynecologists and the proportion of laparoscopic surgeries for benign gynecological diseases in Japan: An ecological study	Open data	Socioeconomic comparison	Health policy evaluation and utilization	Neoplasms		benign gynecological diseases	No age limit/ Others		
Isobe 2020	The number of overall hysterectomies per population with the perimenopausal status is increasing in Japan: A national representative cohort study	Open data	Medical treatment status	Health policy evaluation and utilization	Neoplasms		hysterectomies for benign gynecologic diseases	Adults	females aged 40 –54 years	
Ito 2022	Regional disparities in cardiac rehabilitation volume throughout Japan based on open data from a National Database of Health Insurance Claims	Open data	Medical treatment status	Socioeconomic comparison	Diseases of the circulatory system		cardiac rehabilitation	No age limit/ Others		
Itoh 2019	National burden of the pharmaceutical cost of wet compresses and its cost predictors: nationwide cross-sectional study in Japan	Open data	Health economics		Others		wet compresses	No age limit/ Others		
Iwamoto 2022	Change in use of pediatric oral antibiotics in Japan, pre- and post-implementation of an antimicrobial resistance action plan	General Data	Medical treatment status	Health policy evaluation and utilization	Others		antimicrobial use	Children	aged ≤19 years	
Iwao 2022	A Survey on Cases of Serious and High-Risk Child Abuse with Trauma Using the Database of Health Insurance Claims	Sampling data	Clinical epidemiology, course of diseases		Injury, poisoning and certain other consequences of external causes		Child abuse	Children	under the age of 18	
Izumi 2019	Epidemiology of Adults and Children Treated for Nontuberculous Mycobacterial Pulmonary Disease in Japan	General Data	Clinical epidemiology, course of diseases	Socioeconomic comparison	Certain infectious and parasitic diseases		nontuberculous mycobacterial pulmonary disease	No age limit/ Others		ID
Jingushi 2019	[The present situation of the fracture treatment in Japan according to the 2nd open data from the National Database of Health Insurance Claims and Specific Health Checkups of Japan]	Open data	Medical treatment status		Injury, poisoning and certain other consequences of external causes		bone fracture	No age limit/ Others		
Jingushi 2021	Low-intensity pulsed ultrasound is frequently used to treat fractures after osteosynthesis in elderly patients: A study using open data from the national database of health insurance claims of Japan	Open data	Medical treatment status		Diseases of the musculoskeletal system and connective tissue	Injury, poisoning and certain other consequences of external causes	fractures after osteosynthesis	No age limit/ Others		
Kajimoto 2020	Patient and National Economic Burden of Dengue in Japan: Results from Japanese National Claims Database	General Data	Health economics		Certain infectious and parasitic diseases		Dengue	No age limit/ Others		
Kajimoto 2020	Clinical Management of Patients with Dengue Infection in Japan: Results from National Database of Health Insurance Claims	General Data	Medical treatment status	Quality of care	Certain infectious and parasitic diseases		Dengue	No age limit/ Others		

ID (Author Year)	Title (*titles translated by author)	Database	Research area/ theme	Research area/ theme-2	Disease	Disease-2	Notes on disease	Age	Notes on age	ID used for analysis
Kamata 2018	Wide difference in biologics usage and expenditure for the treatment of patients with rheumatoid arthritis in each prefecture in Japan analyzed using "National Database of Health Insurance Claims and Specific Health Checkups of Japan"	Open data	Medical treatment status	Socioeconomic comparison	Diseases of the musculoskeletal system and connective tissue		rheumatoid arthritis	No age limit/ Others		
Kamijo 2020	[A Study on Estimating the Extraction of Permanent Teeth by Main Reason, Using the Japanese National Database of Health Insurance Claims and Specific Health Checkups of Japan (NDB Japan) and the Second Survey of Reasons for Permanent Teeth Extraction]	Open data	Clinical epidemiology, course of diseases	Medical treatment status	Diseases of the digestive system		permanent teeth extraction	No age limit/ Others		
Kamitani 2021	Incidence of lower limb amputation in people with and without diabetes: a nationwide 5-year cohort study in Japan	General Data	Clinical epidemiology, course of diseases		Endocrine, nutritional, and metabolic diseases	Injury, poisoning and certain other consequences of external causes	lower limb amputation, diabetes	No age limit/ Others		personal identifiers
Kanaoka 2021	Current Status and Effect of Outpatient Cardiac Rehabilitation After Percutaneous Coronary Intervention in Japan	General Data	Patient health service utilization	Intervention effect	Diseases of the circulatory system		cardiovascular diseases	Adults	aged ≥20 years	
Kanaoka 2022	Trends and Factors Associated with Cardiac Rehabilitation Participation: Data from Japanese Nationwide Databases	General Data	Patient health service utilization		Diseases of the circulatory system		cardiac rehabilitation	Adults	aged ≥20 years	
Kanaoka 2022	The Impact of Hospital Case Volume on the Outcomes after Catheter Ablation for Atrial Fibrillation according to the Ablation Technology	General Data	Health policy evaluation and utilization		Diseases of the circulatory system		atrial fibrillation	Adults	younger than 20 years and were excluded	
Kanaoka 2022	Multifactorial Effects of Outpatient Cardiac Rehabilitation in Patients with Heart Failure: A Nationwide Retrospective Cohort Study	General Data	Intervention effect		Diseases of the circulatory system		heart failure, cardiac rehabilitation	Adults	aged ≥20 years	
Kanaoka 2022	Hospital- and Patient-level Analysis of Quality Indicators in Acute Coronary Syndrome Care: A Nationwide Database Study	General Data	Quality of care	Intervention effect	Diseases of the circulatory system		acute coronary syndrome	Adults	aged ≥20 years	unique identifier with a patient matching technique (ID0)
Kanaoka 2022	Outpatient cardiac rehabilitation dose after acute coronary syndrome in a nationwide cohort	General Data	Medical treatment status	Intervention effect	Diseases of the circulatory system		acute coronary syndrome, cardiac rehabilitation	Adults	aged ≥20 years	personal identification
Kanaoka 2023_Sodium-Glucose	Sodium-Glucose Cotransporter 2 Inhibitor Use in Early-Phase Acute Coronary Syndrome with Severe Heart Failure	General Data	Intervention effect		Diseases of the circulatory system		acute coronary syndrome	Adults	aged ≥20 years	
Kashima 2022	The 2018 Japan Floods Increased Prescriptions of Antidementia Drugs Among Disaster Victims	General Data	Medical treatment status	Others	Mental, Behavioral and Neurodevelopmental disorders	Diseases of the nervous system	dementia	Older persons	aged ≥65 years	
Katano 2018	Trends in isolated meniscus repair and meniscectomy in Japan, 2011-2016	Open data	Medical treatment status		Diseases of the musculoskeletal system and connective tissue		meniscus repair and meniscectomy	No age limit/ Others		
Katano 2020	Trends in arthroplasty in Japan by a complete survey, 2014-2017	Open data	Medical treatment status	Socioeconomic comparison	Diseases of the musculoskeletal system and connective tissue		arthroplasty	No age limit/ Others		

ID (Author Year)	Title (*titles translated by author)	Database	Research area/ theme	Research area/ theme-2	Disease	Disease-2	Notes on disease	Age	Notes on age	ID used for analysis
Kato 2022	Association between the number of board-certified physiatrists and volume of rehabilitation provided in Japan: an ecological study	Open data	Health policy evaluation and utilization		Others		rehabilitation services	No age limit/ Others		
Kido 2020	Nationwide incidence of central retinal artery occlusion in Japan: an exploratory descriptive study using the National Database of Health Insurance Claims (2011-2015)	Sampling data	Clinical epidemiology, course of diseases		Diseases of the eye and adnexa		central retinal artery occulusion	No age limit/ Others		
Kido 2022	Incidence of central serous chorioretinopathy (2011-2018): a nationwide population-based cohort study of Japan	General Data	Clinical epidemiology, course of diseases	Medical treatment status	Diseases of the eye and adnexa		central serous chorioretinopathy	Adults	aged 30 years or older	ID0
Kido 2022	Incidence and Clinical Practice of Exudative Age-related Macular Degeneration: A Nationwide Population-Based Cohort Study	General Data	Clinical epidemiology, course of diseases	Medical treatment status	Diseases of the eye and adnexa		active exudative age-related macular degeneration	Adults	aged 40 years or older	unique identification
Kimura 2015	[Same Examinations in Different Healthcare Providers in the Same Month of Referral, Analysis by Reimbursement Claim Database]	Sampling data	Medical treatment status		Others		same examinations in different healthcare provider	No age limit/ Others		
Kinoshita 2019	Nationwide study of outpatient oral antimicrobial utilization patterns for children in Japan (2013-2016)	General Data	Medical treatment status	Health policy evaluation and utilization	Others		antimicrobial consumption	Children	<15 years of age	
Kitazawa 2017	Cost Analysis of Transplantation in Japan, Performed With the Use of the National Database	General Data	Health economics		Others		Transplantation	No age limit/ Others		
Kobayashi 2022	Risks of Myocarditis and Pericarditis Following Vaccination with SARS-CoV-2 mRNA Vaccines in Japan: An Analysis of Spontaneous Reports of Suspected Adverse Events	General Data	Intervention effect		Certain infectious and parasitic diseases	Diseases of the circulatory system	COVID-19, myocarditis or pericarditis	No age limit/ Others		ID0
Kodama 2021	Are Public Oral Care Services Evenly Distributed?-Nation-Wide Assessment of the Provision of Oral Care in Japan Using the National Database of Health Insurance Claims	General Data	Medical treatment status	Socioeconomic comparison	Diseases of the digestive system		oral health care services	No age limit/ Others		
Koizumi 2021	Effect of population inflow and outflow between rural and urban areas on regional antimicrobial use surveillance	Accumulated data	Medical treatment status	Socioeconomic comparison	Others		antimicrobial use	No age limit/ Others		
Koizumi 2023	Effects of population age structure on parenteral antimicrobial use estimations	General Data	Medical treatment status	Research methodology	Others		antimicrobial use	No age limit/ Others		
Komada 2017	[Surveillance to Determine Adverse Reactions to Carbamazepine and Lamotrigine: Analysis of the "Japanese Adverse Drug Event Report", "Information on Adverse Reaction Relief Benefits" and "Health] Insurance Claims and Specific Health Checkups of Japan" Databases]	Open data	Intervention effect		Diseases of the nervous system		antiepileptic drug therapy	No age limit/ Others		
Komamine 2019	Cardiovascular risks associated with dipeptidyl peptidase-4 inhibitors monotherapy compared with other antidiabetes drugs in the Japanese population: A nationwide cohort study	General Data	Intervention effect		Endocrine, nutritional, and metabolic diseases	Diseases of the circulatory system	diabetes, cardiovascular disease	No age limit/ Others		
Komiyama 2023	Hospital-Level Variation in Cardiac Rehabilitation After Myocardial Infarction in Japan During Fiscal Years 2014-2015 Using the National Database	General Data	Patient health service utilization	Health policy evaluation and utilization	Diseases of the circulatory system		acute myocardial infarction, cardiac rehabilitation	Adults	aged ≥20 years	
Koyama 2017	Patterns of CT use in Japan, 2014: A nationwide cross-sectional study	Open data	Socioeconomic comparison	Medical treatment status	Others		CT examinations	No age limit/ Others		
Koyama 2021	[Real situation of Cardiac Rehabilitation from the Viewpoint of Medical service fees]	Open data	Medical treatment status		Diseases of the circulatory system		cardiac rehabilitation	No age limit/ Others		
Koyama 2022	[Real Situation of Rehabilitation Therapy from the Viewpoint of Medical Service Fees]	Open data	Medical treatment status		Others		rehabilitation therapy	No age limit/ Others		
Kubo 2017	[The need and key points for patient matching in clinical studies using the National Database of Health Insurance Claims and Specific Health Checkups of Japan (NDB)]	General Data	Research methodology		Others		patient matching	No age limit/ Others		ID1,ID2

ID (Author Year)	Title (*titles translated by author)	Database	Research area/ theme	Research area/ theme-2	Disease	Disease-2	Notes on disease	Age	Notes on age	ID used for analysis
Kubo 2021	Tracing all patients who received insured dialysis treatment in Japan and the present situation of their number of deaths	General Data	Research methodology		Diseases of the genitourinary system		dialysis	No age limit/ Others		
Kubo 2021	[Mortality Tracking using the National Database of Health Insurance Claims and Specific Health Checkups of Japan (NDB)]	General Data	Research methodology		Others		mortality tracking	Adults	aged ≥ 40 years	
Kubota 2015	Epidemiology of psoriasis and palmoplantar pustulosis: a nationwide study using the Japanese national claims database	General Data	Clinical epidemiology, course of diseases		Diseases of the skin and subcutaneous tissue		psoriasis and palmoplantar pustulosis	No age limit/ Others		ID1 and ID2 pairs
Kubota 2022	Effectiveness and Safety of Reduced and Standard Daily Doses of Direct Oral Anticoagulants in Patients with Nonvalvular Atrial Fibrillation: A Cohort Study Using National Database Representing the Japanese Population	General Data	Medical treatment status	Intervention effect	Diseases of the circulatory system		nonvalvular atrial fibrillation	Adults	at least 20 years old	ID1 and ID2
Kumamaru 2018	Global and Japanese regional variations in radiologist potential workload for computed tomography and magnetic resonance imaging examinations	Open data	Socioeconomic comparison	Others	Others		CT and MRI examinations performed	No age limit/ Others		
Kuniyoshi 2021	Regional variation in the development of neonatal hyperbilirubinemia and relation with sunshine duration in Japan: an ecological study	Open data	Socioeconomic comparison	Clinical epidemiology, course of diseases	Certain conditions originating in the perinatal period		neonatal hyperbilirubinemia	Children	neonate	
Kuramochi 2022	Drug Combinations for Mood Disorders and Physical Comorbidities That Need Attention: A Cross-Sectional National Database Survey	Sampling data	Medical treatment status		Mental, Behavioral and Neurodevelopmental disorders		mood disorders	No age limit/ Others		
Kurobe 2022	[Trends in the inpatient rehabilitation practices in Japan : NDB open data analysis]	Open data	Medical treatment status	Socioeconomic comparison	Others		inpatient rehabilitation	No age limit/ Others		
Kurosaki 2020	[Medical expenses for diabetes care in Japan: Analysis of inter-prefecture differences]	Open data	Health economics	Socioeconomic comparison	Endocrine, nutritional, and metabolic diseases		diabetes	Adults	≥40 years of age	
Kusama 2021	Characteristics and limitations of national antimicrobial surveillance according to sales and claims data	General Data	Medical treatment status	Research methodology	Others		antimicrobial use	No age limit/ Others		
Kuwajima 2023	[Investigation of Orthognathic Surgery in Japan Using the National Database]	General Data	Medical treatment status		Diseases of the digestive system		jaw deformity	No age limit/ Others		ID0v2
Kuwata 2022	Association between dipeptidyl peptidase-4 inhibitors and increased risk for bullous pemphigoid within 3 months from first use: A 5-year population-based cohort study using the Japanese National Database	General Data	Intervention effect		Diseases of the skin and subcutaneous tissue	Endocrine, nutritional, and metabolic diseases	bullous pemphigoid, DPP-4is	No age limit/ Others		ID0
Maeda 2018	Cesarean section rates and local resources for perinatal care in Japan: A nationwide ecological study using the national database of health insurance claims	Accumulated data	Medical treatment status	Socioeconomic comparison	Pregnancy, childbirth and the puerperium		cesarean delivery	No age limit/ Others		
Maeda 2021	Regional Disparity of Reperfusion Therapy for Acute Ischemic Stroke in Japan: A Retrospective Analysis of Nationwide Claims Data from 2010 to 2015	General Data	Socioeconomic comparison	Medical treatment status	Diseases of the circulatory system		acute ischemic stroke	No age limit/ Others		
Maeda 2021	Nationwide temporal trend analysis of reperfusion therapy utilization and mortality in acute ischemic stroke patients in Japan	General Data	Medical treatment status	Intervention effect	Diseases of the circulatory system		acute ischemic stroke	No age limit/ Others		
Maeda 2021	Cesarean delivery rates for overall and multiple pregnancies in Japan: A descriptive study using nationwide health insurance claims data	General Data	Medical treatment status	Socioeconomic comparison	Pregnancy, childbirth and the puerperium		cesarean delivery	No age limit/ Others		ID1 and ID2
Mamiya 2022	Impact of reimbursement restriction on drug market sales under the National Health Insurance in Japan	Open data	Health economics	Health policy evaluation and utilization	Others		drug market sales	No age limit/ Others		

ID (Author Year)	Title (*titles translated by author)	Database	Research area/ theme	Research area/ theme-2	Disease	Disease-2	Notes on disease	Age	Notes on age	ID used for analysis
Matsubayashi 2020	Prevalence, incidence, comorbidities, and treatment patterns among Japanese patients with acromegaly: a descriptive study using a nationwide claims database	General Data	Clinical epidemiology, course of diseases	Medical treatment status	Endocrine, nutritional, and metabolic diseases		acromegary	Adults	≥20 years of age	ID1 or ID2 matched
Matsuda 2015	Analysis of Health Care Region for Psychiatric Care Based on the National Database	General Data	Health policy evaluation and utilization		Mental, Behavioral and Neurodevelopmental disorders		Psychiatric medicine	No age limit/ Others		ID
Matsuda 2015	Analysis of Disease Structure for the Regional Health Care Plan Based on the National Database	General Data	Health policy evaluation and utilization		Diseases of the circulatory system		cerebral infarction patiens	No age limit/ Others		ID
Matsuda 2023	[Epidemiological Study of Dupuytren's Contracture in Japan]	Open data	Clinical epidemiology, course of diseases		Diseases of the musculoskeletal system and connective tissue		Dupuytren's Contracture	No age limit/ Others		
Matsumoto 2018	Relationship between the Number of Adult Obesity and Neuropsychiatric Prescription Drugs	Open data	Clinical epidemiology, course of diseases	Medical treatment status	Endocrine, nutritional, and metabolic diseases	Mental, Behavioral and Neurodevelopmental disorders	obesity and neuropsychiatric	Adults	aged 40-74 years	
Matsuoka 2023	Projected numbers of knee and hip arthroplasties up to the year 2030 in Japan	Open data	Prediction model	Medical treatment status	Diseases of the musculoskeletal system and connective tissue		osteoarthritis	Adults	aged 40 years and over	
Mihara 2020	Factors correlated with drug use for constipation: perspectives from the 2016 open Japanese National Database	Open data	Medical treatment status	Clinical epidemiology, course of diseases	Diseases of the digestive system		constipation	Adults	between 40 and 74 years old	
Minamizono 2019	[Gender, age, and regional distribution of equivalent conversion values for outpatient prescription psychotropic drugs: From the 2nd NDB Open Database]*	Open data	Medical treatment status	Socioeconomic comparison	Mental, Behavioral and Neurodevelopmental disorders		psychotropics	No age limit/ Others		
Mita 2021	An alternative index for evaluating AMU and anti-methicillin-resistant Staphylococcus aureus agent use: A study based on the National Database of Health Insurance Claims and Specific Health Checkups data of Japan	Accumulated data	Medical treatment status	Research methodology	Others		antimicrobial use	No age limit/ Others		
Mitsutake 2019	[Study on International Statistical Indicators using the National Database of Health Insurance Claims and Specific Health Checkups of Japan]	General Data	Medical treatment status	Health policy evaluation and utilization	Others		medical treatment information	No age limit/ Others		
Miyashita 2022	Changes in the Characteristics and Outcomes of COVID-19 Patients from the Early Pandemic to the Delta Variant Epidemic: A Nationwide Population- based Study	General Data	Clinical epidemiology, course of diseases		Certain infectious and parasitic diseases		COVID-19	Adults	adult patients	
Mizukami 2023	Disease trends after Helicobacter pylori eradication based on Japanese nationwide claims and the health check-up database	General Data	Intervention effect		Certain infectious and parasitic diseases	Diseases of the musculoskeletal system and connective tissue	Helicobacter pylori	No age limit/ Others		
Mizuno 2022	Search for indexes to evaluate trends in antibiotic use in the sub-prefectural regions using the National Database of Health Insurance Claims and Specific Health Checkups of Japan	Accumulated data	Medical treatment status	Research methodology	Others		antimicrobial use	No age limit/ Others		
Mizuno 2021	Differences in aggressive treatments during the actively dying phase in patients with cancer and heart disease: an exploratory study using the sampling dataset of the National Database of Health Insurance Claims	Sampling data	Medical treatment status		Neoplasms	Diseases of the circulatory system	cancer and heart disease (aggressive treatments)	No age limit/ Others		
Mohri 2023	Risk of Lactic Acidosis in Hospitalized Diabetic Patients Pre-scribed Biguanides in Japan: A Retrospective Total-Population Cohort Study	General Data	Intervention effect		Endocrine, nutritional, and metabolic diseases		diabetes mellitus	No age limit/ Others		ID0

ID (Author Year)	Title (*titles translated by author)	Database	Research area/ theme	Research area/ theme-2	Disease	Disease-2	Notes on disease	Age	Notes on age	ID used for analysis
Mori 2022	Medical expenditures for fragility hip fracture in Japan: a study using the nationwide health insurance claims database	General Data	Health economics		Injury, poisoning and certain other consequences of external causes	Diseases of the musculoskeletal system and connective tissue	fragility hip fracture	Older persons	aged 60 years and older	ID0
Morii 2019	Projecting future supply and demand for physical therapists in Japan using system dynamics	Open data	Prediction model	Health policy evaluation and utilization	Others		supply and demand for physical therapists	Adults	aged ≥15 years	
Morita 2022	[Future design based on estimation of the demand and supply of radiological technicians using real-world data]*	Open data	Prediction model	Health policy evaluation and utilization	Others		diagnostic imaging	No age limit/ Others		
Mukai 2020	Trends Associated with Hemorrhoids in Japan: Data Mining of Medical Information Datasets and the National Database of Health Insurance Claims and Specific Health Checkups of Japan (NDB) Open Data Japan	Open data	Medical treatment status	Clinical epidemiology, course of diseases	Diseases of the digestive system		hemorrhoids	No age limit/ Others		
Muro 2023	Utility of Blood Culture in Patients with Community-Acquired Pneumonia: A Propensity Score-Matched Analysis Based on a Japanese National Health Insurance Database	General Data	Medical treatment status	Intervention effect	Certain infectious and parasitic diseases	Diseases of the respiratory system	community-acquired pneumonia	Adults	aged >15 years	
Muronaga 2022	Lithium in drinking water and Alzheimer's dementia: Epidemiological Findings from National Data Base of Japan	General Data	Clinical epidemiology, course of diseases		Diseases of the nervous system		Alzheimer's dementia(AD)	Older persons	aged 65 years or older	
Myojin 2022	Development of a New Method to Trace Patient Data Using the National Database in Japan	General Data	Research methodology		Others		method to trace patient data	No age limit/ Others		ID0
Nagakura 2022	Lifestyle habits to prevent the development of benign prostatic hyperplasia: Analysis of Japanese nationwide datasets	Open data	Clinical epidemiology, course of diseases		Diseases of the genitourinary system		benign prostatic hyperplasia	Adults	aged 40–74 years	
Naito 2021	Comorbidities and co-medications among 28089 people living with HIV: A nationwide cohort study from 2009 to 2019 in Japan	General Data	Clinical epidemiology, course of diseases	Medical treatment status	Certain infectious and parasitic diseases		HIV, PLWH	No age limit/ Others		
Naito 2022	Analysis of antiretroviral therapy switch rate and switching pattern for people living with HIV from a national database in Japan	General Data	Medical treatment status	Intervention effect	Certain infectious and parasitic diseases		HIV, PLWH	No age limit/ Others		
Naito 2022	Syphilis in people living with HIV does not account for the syphilis resurgence in Japan	General Data	Clinical epidemiology, course of diseases		Certain infectious and parasitic diseases		HIV, syphilis	No age limit/ Others		
Nakai 2022	Contemporary use of SGLT2 inhibitors in heart failure patients with diabetes mellitus: A comparison of DPP4 inhibitors in a nationwide electric health database of the superaged society	General Data	Intervention effect		Diseases of the circulatory system	Endocrine, nutritional, and metabolic diseases	heart failure patients with diabetes mellitus	No age limit/ Others		ID0
Nakai 2022	Age-dependent Association of Discharge Heart- Failure Medications with Clinical Outcomes in a Super-aged Society	General Data	Socioeconomic comparison	Intervention effect	Diseases of the circulatory system		acute heart-failure	No age limit/ Others		ID0
Nakajima 2020	Prevalence of patients with rheumatoid arthritis and age-stratified trends in clinical characteristics and treatment, based on the National Database of Health Insurance Claims and Specific Health Checkups of Japan	General Data	Clinical epidemiology, course of diseases	Medical treatment status	Diseases of the musculoskeletal system and connective tissue		rheumatoid arthritis	Adults	aged ≥16 years	ID1
Nakajima 2021	Geographic variations in rheumatoid arthritis treatment in Japan: A nationwide retrospective study using the national database of health insurance claims and specific health checkups of Japan	General Data	Medical treatment status	Socioeconomic comparison	Diseases of the musculoskeletal system and connective tissue		rheumatoid arthritis	Adults	16 years old or older	
Nakamura 2015	[Evaluation of the Number of Varicella Patients Estimated by Prescription Surveillance]	Accumulated data	Clinical epidemiology, course of diseases	Research methodology	Certain infectious and parasitic diseases		varicella	No age limit/ Others		
Nakamura 2015	[Evaluation of the estimated number of influenza patients by prefecture in pharmacy surveillance using nationwide electronic medical health insurance claims]*	Accumulated data	Clinical epidemiology, course of diseases	Research methodology	Diseases of the respiratory system		influenza	No age limit/ Others		

ID (Author Year)	Title (*titles translated by author)	Database	Research area/ theme	Research area/ theme-2	Disease	Disease-2	Notes on disease	Age	Notes on age	ID used for analysis
Nakamura 2015	Evaluation of estimated number of influenza patients from national sentinel surveillance using the national database of electronic medical claims	Accumulated data	Clinical epidemiology, course of diseases	Research methodology	Diseases of the respiratory system		influenza	No age limit/ Others		
Nakamura 2015	Proposition of real-time precise prediction model of infectious disease patients from Prescription Surveillance using the National Database of Electronic Medical Claims	Accumulated data	Prediction model	Clinical epidemiology, course of diseases	Certain infectious and parasitic diseases		infectious disease	No age limit/ Others		
Nakamura 2018	Severe abnormal behavior incidence after administration of neuraminidase inhibitors using the national database of medical claims	General Data	Intervention effect		Diseases of the respiratory system		influenza	Children	5-19years	
Nakanishi 2022	The Use of Topical Antibiotics Based on the National Database of Health Insurance Claims and Specific Health Checkups of Japan (NDB) Open Data in 2017	Open data	Medical treatment status		Others		topical antimicrobials	No age limit/ Others		
Nakatoh 2021	Insufficient increase in bone mineral density testing rates and pharmacotherapy after hip and vertebral fracture: analysis of the National Database of Health Insurance Claims and Specific Health Checkups of Japan	General Data	Medical treatment status	Socioeconomic comparison	Injury, poisoning and certain other consequences of external causes	Diseases of the musculoskeletal system and connective tissue	hip and vertebral fracture, osteoporosis	Adults	aged ≥ 50 years	
Nakatoh 2021	Insufficient persistence to pharmacotherapy in Japanese patients with osteoporosis: an analysis of the National Database of Health Insurance Claims and Specific Health Checkups in Japan	General Data	Patient health service utilization	Socioeconomic comparison	Diseases of the musculoskeletal system and connective tissue		osteoporosis	Adults	aged ≥ 50 years	
Nakatoh 2022	Association of pharmacotherapy with second hip fracture incidence: a retrospective analysis of the National Database of Health Insurance Claims and Specific Health Checkups of Japan	General Data	Intervention effect		Injury, poisoning and certain other consequences of external causes	Diseases of the musculoskeletal system and connective tissue	hip and vertebral fracture, glucocorticoid-induced osteoporosis	Adults	aged ≥50 years	
Nakatoh 2023	Association between pharmacotherapy and secondary hip fracture in a real-world setting: a nationwide database study	General Data	Intervention effect		Diseases of the musculoskeletal system and connective tissue		secondary hip fracture	Adults	aged ≥ 50 years	
Ninomiya 2022	Nationwide comprehensive epidemiological study of rare diseases in Japan using a health insurance claims database	Accumulated data	Clinical epidemiology, course of diseases		Others		rare diseases	No age limit/ Others		
Nishi 2020	Regional variance in the use of urine dipstick test for outpatients in Japan	Open data	Medical treatment status	Socioeconomic comparison	Diseases of the genitourinary system		urine dipstick test	No age limit/ Others		
Nishioka 2020	Incidence and seasonality of type 1 diabetes: a population-based 3-year cohort study using the National Database in Japan	General Data	Clinical epidemiology, course of diseases		Endocrine, nutritional, and metabolic diseases		Type 1 diabetes	No age limit/ Others		ID0
Nishioka 2020	Absolute risk of acute coronary syndrome after severe hypoglycemia: A population-based 2-year cohort study using the National Database in Japan	General Data	Clinical epidemiology, course of diseases		Endocrine, nutritional, and metabolic diseases	Diseases of the circulatory system	Diabetes, acute coronary syndrome	Adults	aged ≥35 years	ID0
Nishioka 2021	Association between influenza and the incidence rate of new-onset type 1 diabetes in Japan	General Data	Clinical epidemiology, course of diseases		Endocrine, nutritional, and metabolic diseases	Diseases of the respiratory system	type 1 diabetes, influenza	No age limit/ Others		ID0
Noda 2017	[Improvements and verification of the patient matching (name matching) method in health insurance claims information and National DataBase (NDB) on specific medical checkups]*	General Data	Research methodology		Others		patient matching	No age limit/ Others		ID0
Noda 2023	Cost-effectiveness analysis of cardiac implantable electronic devices with reactive atrial-based antitachycardia pacing	Open data	Health economics		Diseases of the circulatory system		cardiac implantable electronic devices	No age limit/ Others		
Nojiri 2019	Comorbidity status in hospitalized elderly in Japan: Analysis from National Database of Health Insurance Claims and Specific Health Checkups	General Data	Clinical epidemiology, course of diseases		Others		comorbidity status of hospitalized elderly patients	Older persons	aged ≥60 years	

ID (Author Year)	Title (*titles translated by author)	Database	Research area/ theme	Research area/ theme-2	Disease	Disease-2	Notes on disease	Age	Notes on age	ID used for analysis
Nomura 2021	[Research on regional differences in the medical treatment status and patient health services utilization, and the provision of information]*	General Data	Medical treatment status	Socioeconomic comparison	Others		medical expenses	No age limit/ Others		
Ochibe 2020	[Study of the Effect of the Perioperative Administration of Statin on the Prognosis of Percutaneous Coronary Intervention Using the National Health Insurance Claims Database in Japan]	General Data	Intervention effect		Diseases of the circulatory system		statin administration during PCI and cardiovascular events	No age limit/ Others		ID
Ogawa 2022	[A Survey of 0-year-old Infants with Cleft Lip and Palate Patients in Tohoku Region Using the National Database]	General Data	Patient health service utilization		Congenital malformations, deformations and chromosomal abnormalities		Cleft Lip and Palate	Children	aged <1 year	ID0v2
Ohara 2020	Fracture risk increased by concurrent use of central nervous system agents in older people: Nationwide case-crossover study	General Data	Intervention effect		Diseases of the musculoskeletal system and connective tissue	Injury, poisoning and certain other consequences of external causes	fragility fracture	Older persons	aged ≥65 years	
Ohara 2020	Central Nervous System Agent Classes and Fragility Fracture Risk among Elderly Japanese Individuals in a Nationwide Case-Crossover Design Study	General Data	Intervention effect		Diseases of the musculoskeletal system and connective tissue	Injury, poisoning and certain other consequences of external causes	fragility fracture	Older persons	aged ≥65 years	
Ohtera 2021	A nationwide survey on participation in cardiac rehabilitation among patients with coronary heart disease using health claims data in Japan	General Data	Medical treatment status	Patient health service utilization	Diseases of the circulatory system		coronary heart disease, cardiac rehabilitation	Adults	aged ≥ 40 years	
Oizumi 2021	[Number of Prescriptions for Heparin Analogues Calculated by Year Using NDB Open Data]	Open data	Health policy evaluation and utilization		Diseases of the skin and subcutaneous tissue		heparin analogs	No age limit/ Others		
Okamoto 2021	Japan's dental care facing population aging: How universal coverage responds to the changing needs of the elderly	Open data	Clinical epidemiology, course of diseases	Patient health service utilization	Diseases of the digestive system		dentistry	No age limit/ Others		
Okazaki 2022	Impact of the 2018 Japan Floods on benzodiazepine use: a longitudinal analysis based on the National Database of Health Insurance Claims	General Data	Medical treatment status	Others	Mental, Behavioral and Neurodevelopmental disorders	Diseases of the nervous system	benzodiazepines and benzodiazepine-related drugs	Adults	aged ≥20 years	
Okazaki 2022	Impact of the 2018 Japan Floods on prescriptions for migraine: A longitudinal analysis using the National Database of Health Insurance Claims	General Data	Medical treatment status	Others	Diseases of the nervous system		migraine	Adults	between the ages of 15 and 64 years	
Okazaki 2022	Increased prescriptions for irritable bowel syndrome after the 2018 Japan Floods: a longitudinal analysis based on the Japanese National Database of Health Insurance Claims and Specific Health Checkups	General Data	Medical treatment status	Others	Diseases of the digestive system		irritable bowel syndrome	Adults	younger than 15 years of age were excluded	
Okubo 2020	Impacts of Primary Care Physician System on Healthcare Utilization and Antibiotic Prescription: Difference-in-Differences and Causal Mediation Analyses	General Data	Medical treatment status	Health policy evaluation and utilization	Certain infectious and parasitic diseases	Diseases of the respiratory system	respiratory infections	Children	<2 years of age	ID
Okubo 2022	The consequence of financial incentives for not prescribing antibiotics: a Japan's nationwide quasi-experiment	General Data	Health policy evaluation and utilization		Others		antibiotics	Children	aged <12 months	
Okui 2021	Analysis of the regional difference in the number of multi-drug prescriptions and its predictors in Japan, 2015-2018	Open data	Medical treatment status	Socioeconomic comparison	Others		multi-drug prescriptions	No age limit/ Others		
Okui 2021	An Age-Period-Cohort Analysis of Prevalence and Consultation Rate for Dyslipidemia in Japan	Open data	Clinical epidemiology, course of diseases	Patient health service utilization	Endocrine, nutritional, and metabolic diseases		dyslipidemia	Adults	aged 40-74 years	

ID (Author Year)	Title (*titles translated by author)	Database	Research area/ theme	Research area/ theme-2	Disease	Disease-2	Notes on disease	Age	Notes on age	ID used for analysis
Okui 2022	Analysis of regional differences in the amount of hypnotic and anxiolytic prescriptions in Japan using nationwide claims data	Open data	Medical treatment status	Socioeconomic comparison	Mental, Behavioral and Neurodevelopmental disorders	Diseases of the nervous system	hypnotic and anxiolytic prescriptions	No age limit/ Others		
Okumura 2013	[Antipsychotics prescribing patterns of patients with schizophrenia in Japan : Using the National Database of Health Insurance Claim Information and Specified Medical Checkups]	Sampling data	Medical treatment status	Quality of care	Mental, Behavioral and Neurodevelopmental disorders		schizophrenia	No age limit/ Others		ID1
Okumura 2017	Epidemiology of overdose episodes from the period prior to hospitalization for drug poisoning until discharge in Japan: An exploratory descriptive study using a nationwide claims database	General Data	Clinical epidemiology, course of diseases	Medical treatment status	Mental, Behavioral and Neurodevelopmental disorders	Injury, poisoning and certain other consequences of external causes	overdose episodes	No age limit/ Others		ID1
Okumura 2017	Risk of recurrent overdose associated with prescribing patterns of psychotropic medications after nonfatal overdose	General Data	Intervention effect	Medical treatment status	Mental, Behavioral and Neurodevelopmental disorders	Injury, poisoning and certain other consequences of external causes	psychotropic medications after nonfatal overdose	Adults	19- to 64-year-old	ID1
Okumura 2018	Glucose and prolactin monitoring in children and adolescents initiating antipsychotic therapy	General Data	Medical treatment status		Mental, Behavioral and Neurodevelopmental disorders		antipsychotics	Children	aged ≤18 years	ID0
Okumura 2018	Timely follow-up visits after psychiatric hospitalization and readmission in schizophrenia and bipolar disorder in Japan	General Data	Intervention effect	Patient health service utilization	Mental, Behavioral and Neurodevelopmental disorders		schizophrenia or bipolar disorder	No age limit/ Others	aged<65 years	ID0
Okumura 2018	Association of high psychiatrist staffing with prolonged hospitalization, follow-up visits, and readmission in acute psychiatric units: a retrospective cohort study using a nationwide claims database	General Data	Health policy evaluation and utilization		Mental, Behavioral and Neurodevelopmental disorders		Patients newly admitted to acute psychiatric units	No age limit/ Others		ID0
Okumura 2019	Psychiatric Admissions and Length of Stay During Fiscal Years 2014 and 2015 in Japan: A Retrospective Cohort Study Using a Nationwide Claims Database	General Data	Medical treatment status		Mental, Behavioral and Neurodevelopmental disorders		psychiatric care	No age limit/ Others		ID0
Ono 2018	Utilization of Anticoagulant and Antiplatelet Agents Among Patients With Atrial Fibrillation Undergoing Percutaneous Coronary Intervention - Retrospective Cohort Study Using a Nationwide Claims Database in Japan	General Data	Medical treatment status		Diseases of the circulatory system		Atrial Fibrillation Undergoing Percutaneous Coronary Intervention	No age limit/ Others		
Ono 2020	The first national survey of antimicrobial use among dentists in Japan from 2015 to 2017 based on the national database of health insurance claims and specific health checkups of Japan	Accumulated data	Medical treatment status		Others		antimicrobial use	No age limit/ Others		
Ono 2020	[Age-and gender-specific prescriptions and fracture surgeries of osteoporosis treatment in Japan using the National Health Insurance Claim Database]	Open data	Medical treatment status	Socioeconomic comparison	Diseases of the musculoskeletal system and connective tissue	Injury, poisoning and certain other consequences of external causes	fracture, osteoporosis	Adults	aged ≥50 years	
Ono 2021	Generic drug usage in dentistry across Japan: Analysis using a Japanese national database	Open data	Medical treatment status	Socioeconomic comparison	Diseases of the digestive system		dentistry	No age limit/ Others		
Oshima 2018	[Correlations between regional distribution of dental hygienists and dentistry service : Analysis using NDB open data Japan]	Open data	Medical treatment status	Socioeconomic comparison	Diseases of the digestive system		dentistry service	No age limit/ Others		
Ota 2020	[Proposal of Method for Estimating the Situation of Regional Healthcare Using NDB Open Data and Sickbed Function Reports Data: Estimation of Implementation Status of Surgery in Inpatient Care in Okayama Prefecture]	Open data	Socioeconomic comparison	Medical treatment status	Others		Implementation status of surgery in inpatient care	No age limit/ Others		

ID (Author Year)	Title (*titles translated by author)	Database	Research area/ theme	Research area/ theme-2	Disease	Disease-2	Notes on disease	Age	Notes on age	ID used for analysis
Otsubo 2015	Regional variations in in-hospital mortality, care processes, and spending in acute ischemic stroke patients in Japan	General Data	Medical treatment status	Socioeconomic comparison	Diseases of the circulatory system		ischemic stroke	Older persons	aged ≥65 years	
Saito 2021	Regional variations in the utilization of adaptive servo-ventilation and continuous positive airway pressure in Japan: data from the National Database of Health Insurance Claims and Specific Health Checkups of Japan (NDB) Open Data Japan	Open data	Medical treatment status	Socioeconomic comparison	Diseases of the nervous system		sleep apnea	No age limit/ Others		
Sakakibara 2019	[Study on the Risk Factors for Postoperative Delirium Using the National Health Insurance Claims Database in Japan]	General Data	Clinical epidemiology, course of diseases		Mental, Behavioral and Neurodevelopmental disorders		postoperative delirium	No age limit/ Others		ID
Sakata 2018	Thyroid function tests before prescribing anti-dementia drugs: a retrospective observational study	General Data	Medical treatment status	Quality of care	Mental, Behavioral and Neurodevelopmental disorders	Diseases of the nervous system	dementia	Older persons	aged ≥65 years	
Sato 2016	[A Preliminary Survey to Measure the Quality Indicators of End-of-life Cancer Care Using the Japanese National Database]	Sampling data	Quality of care	Research methodology	Neoplasms		cancer patients	Adults	excluded patients aged <20 years	
Sato 2018	Estimation of total prescription weights of active pharmaceutical ingredients in human medicines based on a public database for environmental risk assessment in Japan	Open data	Medical treatment status		Others		active pharmaceutical ingredients	No age limit/ Others		
Sato 2019	Nationwide survey of severe postpartum hemorrhage in Japan: an exploratory study using the national database of health insurance claims	Sampling data	Clinical epidemiology, course of diseases	Health policy evaluation and utilization	Pregnancy, childbirth and the puerperium		postpartum hemorrhage	No age limit/ Others		
Sato 2021	Disproportionality by sex in the prescription of drugs capable of inducing parkinsonism for the elderly: A survey using statistics of Japanese national health claims from 2014 to 2017	Open data	Medical treatment status	Socioeconomic comparison	Diseases of the nervous system		drug-induced parkinsonism	No age limit/ Others		
Sato 2022	Evaluation of adrenaline auto-injector prescription profiles: A population-based, retrospective cohort study within the National Insurance Claims Database of Japan	General Data	Medical treatment status		Injury, poisoning and certain other consequences of external causes		anaphylaxis	No age limit/ Others		ID variable
Sato 2023	Virtual patient identifier (vPID): Improving patient traceability using anonymized identifiers in Japanese healthcare insurance claims database	General Data	Research methodology		Others		patient traceability	No age limit/ Others		a virtual patient identifier (vPID)
Sawada 2023	Cardiovascular risk of urate-lowering drugs: A study using the National Database of Health Insurance Claims and Specific Health Checkups of Japan	General Data	Intervention effect		Diseases of the musculoskeletal system and connective tissue	Diseases of the circulatory system	urate-lowering drugs and cardiovascular events	Adults	aged ≥20 years	
Sengoku 2022	Prevalence of type 2 diabetes by age, sex and geographical area among two million public assistance recipients in Japan: a cross-sectional study using a nationally representative claims database	Sampling data	Clinical epidemiology, course of diseases	Socioeconomic comparison	Endocrine, nutritional, and metabolic diseases		type 2 diabetes	No age limit/ Others		
Shibata 2014	[Usefulness of a healthcare insurance claims database for statistical data in cancer patients]*	General Data	Research methodology	Clinical epidemiology, course of diseases	Neoplasms		cancer patients	No age limit/ Others		
Shimizu 2018	[A comprehensive survey of clinical practice concerning telemedicine treatment]	Open data	Research methodology		Others		telemedicine treatment	No age limit/ Others		
Shimokawa 2022	[Current status of pediatric sedation for MRI examinations in Japan : the National Database Open Data and random Sampling Data analysis of outpatient clinics]	Sampling data	Medical treatment status		Others		pediatric sedation for MRI examinations	Children	aged <10 years	

ID (Author Year)	Title (*titles translated by author)	Database	Research area/ theme	Research area/ theme-2	Disease	Disease-2	Notes on disease	Age	Notes on age	ID used for analysis
Sugawara 2019	Association of severe abnormal behavior and acetaminophen with/without neuraminidase inhibitors	General Data	Intervention effect		Diseases of the respiratory system		influenza	Children	5-19 year of age	
Sugiyama 2019	Variation in process quality measures of diabetes care by region and institution in Japan during 2015-2016: An observational study of nationwide claims data	General Data	Quality of care	Socioeconomic comparison	Endocrine, nutritional, and metabolic diseases		diabetes	Adults	20 years or older	ID1
Sun 2022	Association between types of home healthcare and emergency house calls, hospitalization, and end- of-life care in Japan	General Data	Medical treatment status		Others		home care support clinics/hospitals	Older persons	aged 65 years or older	
Sun 2022	The effect of home care support clinics on hospital readmission in heart failure patients in Japan	General Data	Intervention effect		Diseases of the circulatory system		heart failure	Older persons	aged ≥65 years	identification numbers
Suwanai 2020	Dipeptidyl Peptidase-4 Inhibitor Reduces the Risk of Developing Hypertrophic Scars and Keloids following Median Sternotomy in Diabetic Patients: A Nationwide Retrospective Cohort Study Using the National Database of Health Insurance Claims of Japan	General Data	Intervention effect		Diseases of the skin and subcutaneous tissue		hypertrophic scars or keloids	No age limit/ Others		
Suzuki 2020	Evaluation of tooth loss among patients with diabetes mellitus using the National Database of Health Insurance Claims and Specific Health Checkups of Japan	General Data	Clinical epidemiology, course of diseases		Endocrine, nutritional, and metabolic diseases	Diseases of the digestive system	tooth loss among patients with diabetes mellitus	Adults	aged 50–74 years	ID1
Suzuki 2021	A Validation Study of the National Database of Health Insurance Claims and Specific Health Checkups of Japan Regarding the Annual Number of Tooth Extractions Performed Across the Entire Japanese Population	Open data	Research methodology		Diseases of the digestive system		tooth extractions	No age limit/ Others		
Suzuki 2022	[Factors related to femoral neck fracture in prefectures : an ecological study using the national database]	Open data	Clinical epidemiology, course of diseases		Injury, poisoning and certain other consequences of external causes		femoral neck fracture	Adults	aged ≥ 40 years	
Suzuki 2023	Promoting generic drug usage in Japan: correlation between generic drug usage and monthly personal income	Open data	Medical treatment status	Socioeconomic comparison	Others		generic drug usage	No age limit/ Others		
Taira 2021	Regional Inequality in Dental Care Utilization in Japan: An Ecological Study Using the National Database of Health Insurance Claims	Open data	Patient health service utilization	Socioeconomic comparison	Diseases of the digestive system		dental care	No age limit/ Others		
Takabayashi 2021	Trend in prescription and treatment retention of molecular-targeted drugs in 121,131 Japanese patients with rheumatoid arthritis: A population- based real-world study	General Data	Medical treatment status	Patient health service utilization	Diseases of the musculoskeletal system and connective tissue		rheumatoid arthritis	No age limit/ Others		ID1 and ID2
Takabayashi 2022	Incidence of opportunistic infections in patients with rheumatoid arthritis treated with different molecular-targeted drugs: A population-based retrospective cohort study	General Data	Intervention effect		Diseases of the musculoskeletal system and connective tissue	Certain infectious and parasitic diseases	rheumatoid arthritis, opportunistic infections	No age limit/ Others		
Takada 2013	[Study on Risk of Gastrointestinal Complications in Low-dose Aspirin Therapy Using the National Receipt Database]	General Data	Intervention effect		Diseases of the digestive system		Gastrointestinal Complications, Low-dose Aspirin Therapy	No age limit/ Others		ID2
Takeshita 2021	[Study on factors related to hospitalization period of acute cerebral infarction using NDB sampling data set]	Sampling data	Clinical epidemiology, course of diseases		Diseases of the circulatory system		acute cerebral infraction	No age limit/ Others		
Takeuchi 2021	Sodium-glucose cotransporter-2 inhibitors and the risk of urinary tract infection among diabetic patients in Japan: Target trial emulation using a nationwide administrative claims database	General Data	Intervention effect		Endocrine, nutritional, and metabolic diseases	Diseases of the genitourinary system	Diabetes, urinary tract infection (UTI)	Adults	aged ≥40 years	

ID (Author Year)	Title (*titles translated by author)	Database	Research area/ theme	Research area/ theme-2	Disease	Disease-2	Notes on disease	Age	Notes on age	ID used for analysis
Tamaki 2019	Estimates of hip fracture incidence in Japan using the National Health Insurance Claim Database in 2012–2015	Accumulated data	Clinical epidemiology, course of diseases	Socioeconomic comparison	Injury, poisoning and certain other consequences of external causes		hip fracture	Adults	aged ≥40 years	
Tamaki 2023	Hip and vertebral fracture risk after initiating antidiabetic drugs in Japanese elderly: a nationwide study	General Data	Intervention effect		Endocrine, nutritional, and metabolic diseases	Injury, poisoning and certain other consequences of external causes	hip and vertebral fracture, diabetes mellitus	Older persons	Patients aged ≥ 65 years	
Tanaka 2017	[Actual use of direct oral anticoagulant (DOAC) in fiscal year 2014 : using the national database of health insurance claims and specific health checkups of Japan (NDB) open data]	Open data	Medical treatment status		Diseases of the circulatory system		actual use of direct oral anticoagulant	Adults	aged ≥20 years	
Tanaka 2018	[Survey of Prescription Volume and Adverse Events of NSAIDs Patches in Fiscal Year 2015]	Open data	Medical treatment status		Others		NSAIDs Patches	No age limit/ Others		
Tanaka 2019	[Investigation of Prescription Pattern of Antiretroviral Using the National Database of Health Insurance Claims Specific Health Checkups of Japan Open Data]	Open data	Medical treatment status		Certain infectious and parasitic diseases		Prescription Pattern of Antiretroviral	Adults	≥20 year of age	
Tanaka 2020	[Survey on prescribing status of NSAIDs patch formulations: Perspectives on promotion of generic drug use and prescribing restrictions]*	Open data	Medical treatment status		Others		NSAIDs	No age limit/ Others		
Tanaka 2022	Understanding the Actual Use of Anti-HIV Drugs in Japan from 2016 to 2019: Demonstrating Epidemiological Relevance of NDB Open Data Japan for Understanding Japanese Medical Care	Open data	Medical treatment status		Certain infectious and parasitic diseases		anti-HIV drug use	No age limit/ Others		
Tanaka 2022	Burden of chronic hepatitis B and C infections in 2015 and future trends in Japan: A simulation study	General Data	Clinical epidemiology, course of diseases	Prediction model	Certain infectious and parasitic diseases		chronic hepatitis B and C infections	No age limit/ Others		
Tanito 2022	Nation-Wide Analysis of Glaucoma Medication Prescription in Fiscal Year of 2019 in Japan	Open data	Medical treatment status		Diseases of the eye and adnexa		antiglaucoma medications	No age limit/ Others		
Tarasawa 2023	Associations Between Death at Home with Medical Resources and Medical Activities in Cancer Patients: A Nationwide Study Using Japanese National Database	Accumulated data	Clinical epidemiology, course of diseases		Neoplasms		death at home among cancer patients	Older persons	aged 65 years and older	
Togashi 2023	Regional Disparities in Transvenous Lead Extraction for Cardiac Implantable Electronic Device Infection in Japan - A Descriptive Study Using the National Database Open Data	Open data	Medical treatment status	Socioeconomic comparison	Diseases of the circulatory system	Injury, poisoning and certain other consequences of external causes	transvenous lead extraction for cardiac implantable electronic device infection	No age limit/ Others		
Tohkin 2016	[Appropriate prescription pattern of medications at the special population]	Sampling data	Medical treatment status	Quality of care	Diseases of the genitourinary system	Diseases of the circulatory system	chronic kidney disease, cardiovascular disease	No age limit/ Others		
Tomic 2022	Lifetime risk, life expectancy, and years of life lost to type 2 diabetes: a multi-national population- based study of 23 high-income jurisdictions	General Data	Clinical epidemiology, course of diseases		Endocrine, nutritional, and metabolic diseases		type 2 diabetes	No age limit/ Others		
Tomida 2023	Statin persistence and adherence among older initiators: A nationwide cohort study using the national health insurance claims database in Japan	General Data	Patient health service utilization		Others		statin use	Adults	aged ≥55 years	
Toyokawa 2017	Estimation of the number of children with cerebral palsy using nationwide health insurance claims data in Japan	General Data	Clinical epidemiology, course of diseases		Diseases of the nervous system		cerebral palsy	Children	aged below 20 years	ID1
Tsuda 2017	Effect on Helicobacter pylori eradication therapy against gastric cancer in Japan	Open data	Intervention effect	Health policy evaluation and utilization	Neoplasms		gastric cancer	No age limit/ Others		

ID (Author Year)	Title (*titles translated by author)	Database	Research area/ theme	Research area/ theme-2	Disease	Disease-2	Notes on disease	Age	Notes on age	ID used for analysis
Tsuji 2023	Trend of anticoagulant therapy in elderly patients with atrial fibrillation considering risks of cerebral infarction and bleeding	Sampling data	Medical treatment status		Diseases of the circulatory system		anticoagulant therapy in patients with non-valvular atrial fibrillation	Older persons	aged ≥ 65 years	
Tsukamoto 2019	Future Perspectives for the Treatment of Diabetes: Importance of a Regulatory Framework	General Data	Medical treatment status		Endocrine, nutritional, and metabolic diseases		diabetes	No age limit/ Others		
Tsuneishi 2016	[Association between Number of Teeth and Medical and Dental Care Expenditure: Analysis Using the Receipt and Health Checkup Information Database in Japan]	General Data	Health economics		Diseases of the digestive system		periodontal disease	Adults	40 years of age or older	ID
Tsuneishi 2017	[Association between Number of Teeth and Medical Visit due to Aspiration Pneumonia in Older People Using the Receipt and Health Checkup Information Database]	General Data	Clinical epidemiology, course of diseases		Diseases of the respiratory system	Diseases of the digestive system	Number of Teeth, Aspiration Pneumonia	Older persons	aged 65 or older	ID
Tsuneishi 2019	[The presence of teeth type using the dental notation of periodontitis patients: a cross-sectional study using the receipt and health checkup information database in Japan]	General Data	Clinical epidemiology, course of diseases	Research methodology	Diseases of the digestive system		Periodontitis	Adults	aged ≥20 years	
Tsuneishil 2021	Association between number of teeth and Alzheimer's disease using the National Database of Health Insurance Claims and Specific Health Checkups of Japan	General Data	Clinical epidemiology, course of diseases		Diseases of the digestive system	Diseases of the nervous system	periodontitis, missing teeth, Alzheimer's disease	Older persons	aged 60 years or older	ID1
Tsunoda 2022	Monthly trends and seasonality of hemodialysis treatment and outcomes of newly initiated patients from the national database (NDB) of Japan	General Data	Research methodology	Clinical epidemiology, course of diseases	Diseases of the genitourinary system		hemodialysis	No age limit/ Others		VPID
Uda 2019	Nationwide survey of indications for oral antimicrobial prescription for pediatric patients from 2013 to 2016 in Japan	General Data	Medical treatment status	Health policy evaluation and utilization	Certain infectious and parasitic diseases	Diseases of the respiratory system	respiratory infections	Children	≤15 years of age	
Ueda 2021	Maternal near-miss attributable to haemorrhagic stroke in patients with hypertensive disorders of pregnancy in Japan: A national cohort study	General Data	Clinical epidemiology, course of diseases	Medical treatment status	Pregnancy, childbirth and the puerperium	Diseases of the circulatory system	haemorrhagic stroke occurring in patients with hypertensive disorders of pregnancy	No age limit/ Others		
Ueda 2022	Impact of efforts to prevent maternal deaths due to obstetric hemorrhage on trends in epidemiology and management of severe postpartum hemorrhage in Japan: a nationwide retrospective study	General Data	Health policy evaluation and utilization		Pregnancy, childbirth and the puerperium		obstetric hemorrhage	No age limit/ Others		
Ueda 2022	[A Survey of Orthognathic Surgery in Tohoku Region Using the National Database]	General Data	Medical treatment status		Diseases of the digestive system		orthognathic surgery	No age limit/ Others		ID0v2
Wada 2021	[Calculation Status of All Full Metal Crowns and CAD/CAM Crowns in Crown Restoration of Molars in the Last 10 Years]	Open data	Medical treatment status		Diseases of the digestive system		crown restoration of molars	No age limit/ Others		
Waki 2022	Prevalence of hypertensive diseases and treated hypertensive patients in Japan: A nationwide administrative claims database study	General Data	Clinical epidemiology, course of diseases	Patient health service utilization	Diseases of the circulatory system		hypertensive diseases	No age limit/ Others		vPID
Watanabe 2022	How are new drugs disseminated in Japan? Analysis using the National Database of Health Insurance Claims of Japan	General Data	Medical treatment status		Neoplasms	Endocrine, nutritional, and metabolic diseases	cancer drugs, diabetes drugs	No age limit/ Others		
Yahaba 2021	Antibiotics for hospitalized children with community-acquired pneumonia in Japan: Analysis based on Japanese national database	Sampling data	Medical treatment status		Diseases of the respiratory system	Certain infectious and parasitic diseases	pneumonia	Children	<15 year of age	
Yamaguchi 2021	Increase in Achilles Tendon Rupture Surgery in Japan: Results From a Nationwide Health Care Database	General Data	Clinical epidemiology, course of diseases	Medical treatment status	Injury, poisoning and certain other consequences of external causes		achilles tendon ruptures	Adults	aged <20 years were excluded	

ID (Author Year)	Title (*titles translated by author)	Database	Research area/ theme	Research area/ theme-2	Disease	Disease-2	Notes on disease	Age	Notes on age	ID used for analysis
Yamamoto 2022	Trends in Open vs. Endoscopic Carpal Tunnel Release: A Comprehensive Survey in Japan	Open data	Medical treatment status	Socioeconomic comparison	Diseases of the nervous system		carpal tunnel syndrome	No age limit/ Others		
Yamasaki 2018	The first report of Japanese antimicrobial use measured by national database based on health insurance claims data (2011-2013): comparison with sales data, and trend analysis stratified by antimicrobial category and age group	Accumulated data	Research methodology	Medical treatment status	Others		antimicrobial use	No age limit/ Others		
Yamauchi 2018	[Current status of osteoporosis screening – Relationship with osteoporotic fractures and need for long-term care –]	Open data	Clinical epidemiology, course of diseases	Medical treatment status	Diseases of the musculoskeletal system and connective tissue		osteoporosis	Adults	40-70 years old	
Yamazaki 2021	Antibiotics prescriptions for pneumonia analyzed by claim information in Japan	Sampling data	Medical treatment status	Quality of care	Diseases of the respiratory system	Certain infectious and parasitic diseases	community-acquired pneumonia	Adults	aged ≥15 years	
Yatomi 2020	Prescription patterns of psychotropics in patients receiving synthetic glucocorticoids	Sampling data	Medical treatment status	Intervention effect	Mental, Behavioral and Neurodevelopmental disorders		psychotropic and glucocorticoids	No age limit/ Others		
Yoshida 2023	Effects of 2018 Japan floods on healthcare costs and service utilization in Japan: a retrospective cohort study	General Data	Health economics	Others	Others		healthcare costs and service utilization	No age limit/ Others		
Yoshimi 2018	[Comparison of prefectures using items related to smoking and quitting smoking in the 2nd NDB Open Data]*	Open data	Research methodology	Socioeconomic comparison	Mental, Behavioral and Neurodevelopmental disorders		nicotine dependency	Adults	aged 40-74 years	
Yoshimura 2018	Survey of anticonvulsant drugs and lithium prescription in women of childbearing age in Japan using a public national insurance claims database	Open data	Medical treatment status	Quality of care	Diseases of the nervous system		Anticonvulsant Drugs	Adults	Childbearing age	

Supplementary File 5 PRISMA-ScR Checklist

SECTION	ITEM	PRISMA-ScR CHECKLIST ITEM	REPORTED ON PAGE #
**TITLE**			
Title	1	Identify the report as a scoping review.	Title
**ABSTRACT**			
Structured summary	2	Provide a structured summary that includes (as applicable): background, objectives, eligibility criteria, sources of evidence, charting methods, results, and conclusions that relate to the review questions and objectives.	Abstract
**INTRODUCTION**			
Rationale	3	Describe the rationale for the review in the context of what is already known. Explain why the review questions/objectives lend themselves to a scoping review approach.	p. 3
Objectives	4	Provide an explicit statement of the questions and objectives being addressed with reference to their key elements (e.g., population or participants, concepts, and context) or other relevant key elements used to conceptualize the review questions and/or objectives.	p. 3
**METHODS**			
Protocol and registration	5	Indicate whether a review protocol exists; state if and where it can be accessed (e.g., a Web address); and if available, provide registration information, including the registration number.	NA
Eligibility criteria	6	Specify characteristics of the sources of evidence used as eligibility criteria (e.g., years considered, language, and publication status), and provide a rationale.	p. 4
Information sources*	7	Describe all information sources in the search (e.g., databases with dates of coverage and contact with authors to identify additional sources), as well as the date the most recent search was executed.	p. 4
Search	8	Present the full electronic search strategy for at least 1 database, including any limits used, such that it could be repeated.	Supplementary file 1
Selection of sources of evidence†	9	State the process for selecting sources of evidence (i.e., screening and eligibility) included in the scoping review.	p. 4
Data charting process‡	10	Describe the methods of charting data from the included sources of evidence (e.g., calibrated forms or forms that have been tested by the team before their use, and whether data charting was done independently or in duplicate) and any processes for obtaining and confirming data from investigators.	p. 4
Data items	11	List and define all variables for which data were sought and any assumptions and simplifications made.	pp. 6-7
Critical appraisal of individual sources of evidence§	12	If done, provide a rationale for conducting a critical appraisal of included sources of evidence; describe the methods used and how this information was used in any data synthesis (if appropriate).	NA
Synthesis of results	13	Describe the methods of handling and summarizing the data that were charted.	pp. 6-7
**RESULTS**			
Selection of sources of evidence	14	Give numbers of sources of evidence screened, assessed for eligibility, and included in the review, with reasons for exclusions at each stage, ideally using a flow diagram.	p. 7, Supplementary file 2
Characteristics of sources of evidence	15	For each source of evidence, present characteristics for which data were charted and provide the citations.	pp. 7-8, Table 1
Critical appraisal within sources of evidence	16	If done, present data on critical appraisal of included sources of evidence (see item 12).	NA
Results of individual sources of evidence	17	For each included source of evidence, present the relevant data that were charted that relate to the review questions and objectives.	Supplementary file 4
Synthesis of results	18	Summarize and/or present the charting results as they relate to the review questions and objectives.	pp. 8-14, Figure 1
**DISCUSSION**			
Summary of evidence	19	Summarize the main results (including an overview of concepts, themes, and types of evidence available), link to the review questions and objectives, and consider the relevance to key groups.	pp. 14-15
Limitations	20	Discuss the limitations of the scoping review process.	p. 16
Conclusions	21	Provide a general interpretation of the results with respect to the review questions and objectives, as well as potential implications and/or next steps.	p. 17
**FUNDING**			
Funding	22	Describe sources of funding for the included sources of evidence, as well as sources of funding for the scoping review. Describe the role of the funders of the scoping review.	Title page
